# Friend of the Devil: Negative Social Influences Driving Substance Use Disorders

**DOI:** 10.3389/fnbeh.2022.836996

**Published:** 2022-02-10

**Authors:** Matthew B. Pomrenze, Franciely Paliarin, Rajani Maiya

**Affiliations:** ^1^Department of Psychiatry and Behavioral Sciences, Stanford University, Stanford, CA, United States; ^2^Department of Physiology, Louisiana State University Health Sciences Center, New Orleans, LA, United States

**Keywords:** social defeat, serotonin, kappa opioid receptor, social isolation, substance use disorders, hyperkatifeia

## Abstract

Substance use disorders in humans have significant social influences, both positive and negative. While prosocial behaviors promote group cooperation and are naturally rewarding, distressing social encounters, such as aggression exhibited by a conspecific, are aversive and can enhance the sensitivity to rewarding substances, promote the acquisition of drug-taking, and reinstate drug-seeking. On the other hand, withdrawal and prolonged abstinence from drugs of abuse can promote social avoidance and suppress social motivation, accentuating drug cravings and facilitating relapse. Understanding how complex social states and experiences modulate drug-seeking behaviors as well as the underlying circuit dynamics, such as those interacting with mesolimbic reward systems, will greatly facilitate progress on understanding triggers of drug use, drug relapse and the chronicity of substance use disorders. Here we discuss some of the common circuit mechanisms underlying social and addictive behaviors that may underlie their antagonistic functions. We also highlight key neurochemicals involved in social influences over addiction that are frequently identified in comorbid psychiatric conditions. Finally, we integrate these data with recent findings on (±)3,4-methylenedioxymethamphetamine (MDMA) that suggest functional segregation and convergence of social and reward circuits that may be relevant to substance use disorder treatment through the competitive nature of these two types of reward. More studies focused on the relationship between social behavior and addictive behavior we hope will spur the development of treatment strategies aimed at breaking vicious addiction cycles.

## Introduction

Perhaps the strongest environmental influences over mammalian behavior are social cues and contexts. Prosocial interactions occur in nearly all mammalian species and are critical for development, survival, and reproduction. Interactions within a group facilitate cooperation to achieve common goals, such as finding food or mates, and can be major determinants of an individual’s ultimate success and fitness. Thus, the neural mechanisms that mediate prosocial behaviors have likely been evolutionarily conserved and involve circuits that modulate a range of motivated behaviors. While positive prosocial behaviors facilitate group motivation for procuring life-saving resources, social experience of negative valence can disrupt group dynamics and lead to pathological behaviors that impair survival.

Social environmental influences are strongly correlated with vulnerability to numerous neuropsychiatric conditions, including substance use disorders (SUDs). Social isolation and ridicule are associated with increased drug intake (Aloise-Young and Kaeppner, 2005; Rusby et al., 2005; Pearson et al., 2006), whereas strong family ties and social competence are associated with lower rates of drug use (Barnes and Farrell, 1992; Scheier et al., 1999; Barnes et al., 2000). Bullying, physical abuse, and the resulting psychological trauma are also predisposing factors for developing SUDs (Back et al., 2000; Niedhammer et al., 2011). Episodes of such physical violence often occur outside the drug-taking contest in schools or at the workplace (Niedhammer et al., 2011). In rodent models, repeated, brief episodes of social defeat stress (SDS) or prolonged social isolation during adolescence reliably and robustly escalate consumption of a variety of drugs, including stimulants and alcohol. In addition, the potentiating effects of social stress on drug intake are long-lasting and do not habituate (Covington and Miczek, 2005). Clearly, social context has a profound effect on the development and maintenance of SUDs.

On the other hand, the influence of SUDs on social behavior appears to follow a similar theme. Chronic drug use often results in social avoidance and self-isolation behaviors that largely reinforce the initial drug-taking. These alterations in social behavioral programs are multifaceted, with strong contributions from negative emotional states produced during drug withdrawal (a state termed “hyperkatifeia”; Koob, 2021) and the strong motivation for drug rewards over natural rewards, like social interaction. These vast changes to the social behavioral state of an individual are supported by anecdotal and scientific evidence alike. These observations lead to the prediction that social deficits resulting from chronic drug use are critical determinants of continued drug use and relapse. Yet, the neural underpinnings that drive these changes in social affect are only starting to be described, despite the potential to serve as a point of therapeutic intervention.

Understanding the neurobiology underlying both social behavior and reward-seeking is essential for leveraging the relationship they have with each other to inform treatment. Virtually all addictive substances exert their primary actions by activating mesolimbic dopamine (DA) circuits and inducing DA release in dorsal and ventral striatal regions (Koob and Volkow, 2010). As natural rewards, such as food and social stimuli, also invoke DA activity in these regions, mesolimbic DA circuitry may serve as a candidate convergence point of social and reward behaviors. However, social behavior dynamics are manifold in nature, recruiting many other neural systems and circuits outside the mesolimbic DA pathway. Understanding how social reward and drug-reward compete at the neural circuit and neurochemical levels may be key to applying social factors to treatment efforts. As emerging data indicate that positive prosocial interactions can buffer addictive behaviors and negative emotional states, innovative strategies that boost sociability, such as therapies focused on social environments or prosocial drugs like MDMA, are of great interest.

In this review, we briefly discuss this vast neurobiology of the social brain and identify convergence points where the interplay of social behaviors and addictive behaviors may be represented in the brain. We review some of the relevant literature describing data on how adverse social environments promote the development of SUDs and how SUDs evoke maladaptive changes in social behavior. Finally, we synthesize these ideas together to identify candidate brain mechanisms that underlie the competitive nature of social and drug-rewards and could inform novel treatment strategies that consider social factors, such as the community reinforcement approach.

## Neurobiology of Prosocial Behaviors

Prosocial behaviors constitute a complex set of social interactions that are essential for development, reproduction, and survival. However, the inability to experience reinforcement from social interaction, or “social reward,” is a hallmark feature of many psychiatric conditions, including SUDs (Chevallier et al., 2012). Early investigations spanning multiple animal models of sociability implicate an important role for DA release in the nucleus accumbens (NAc) in affiliative interactions (Robinson et al., 2002, 2011; Young and Wang, 2004). Recent work confirmed these findings by demonstrating that NAc-projecting ventral tegmental area (VTA) DA neurons show increased activity during prosocial behaviors and optogenetic activation of these cells enhanced sociability (Gunaydin et al., 2014). Considering the general reinforcing nature of DA release in the NAc, these findings are readily predicted. Whether DA has a unique function in social reward compared to other forms of reward is unknown, and whether dopamine drives social approach or merely reinforces social interactions remains to be untangled. Nevertheless, its interactions with other neuromodulators may provide clues to its functions in social contexts.

Young and Wang (2004) identified a critical contribution of the neuropeptide oxytocin in the NAc in mediating pair bonding in prairie voles. The observation that oxytocin potently regulates pair bonding and other affiliative behaviors led to several studies describing this phenomenon. Concurrent activation of both oxytocin and DA D2 receptors in the NAc was found to be a key mechanism mediating prairie vole pair bonding (Liu and Wang, 2003). A recent study demonstrated an interaction between NAc DA and opioid systems in the NAc of prairie voles in maintaining monogamous pair bonds. Pair bonding led to increased D1R and dynorphin mRNA expression along with increased DA release in the NAc, whose signaling mediated aggression towards novel conspecifics and social avoidance (Resendez et al., 2016). Interestingly, alterations in kappa opioid receptor (KOR) function after pair bonding contributed to a neuroprotective effect against amphetamine reward. In addition to NAc, oxytocin signaling in other nodes of the mesolimbic DA pathway influences sociability. Oxytocin is synthesized in parvocellular neurons in the paraventricular hypothalamus (PVN) and these neurons project axons to the NAc and the VTA, among many other regions. Retrograde tracing with rabies viruses revealed monosynaptic connections between VTA DA neurons and PVN oxytocin neurons (Beier et al., 2015). In rats, direct infusion of oxytocin into the VTA increased DA signaling in the NAc and blockade of oxytocin receptors in VTA reduced this effect, as well as pup licking and grooming by dams (Shahrokh et al., 2010). Moreover, oxytocin action in the VTA regulates responses to social cues (Abu-Akel et al., 2016; Buffington et al., 2016; Shamay-Tsoory and Abu-Akel, 2016) and social reward (Song et al., 2016; Hung et al., 2017). Mechanistically, oxytocin promotes social reward by enhancing NAc-projecting VTA DA cell firing (Song et al., 2016; Hung et al., 2017; Xiao et al., 2017). Therefore, oxytocin plays a critical role in promoting social reward by influencing DA activity in the mesolimbic pathway.

Other important neuromodulatory systems besides DA are modulated by oxytocin and critically involved in sociability. The NAc also receives strong inputs from dorsal raphe (DR) serotonin (5-HT) neurons. The role of 5-HT in reward processing is complex, with some studies suggesting control over appetitive responses over long time-scales (Miyazaki et al., 2014; Fonseca et al., 2015; Xu et al., 2017) and some studies showing inhibition of 5-HT activity during reward consumption (McDevitt et al., 2021). As such, 5-HT release is not inherently rewarding like DA (Liu et al., 2020). Despite this complexity, 5-HT’s contributions to social behavior are becoming more and more clear. In mice, social reward learning depends on oxytocin receptor activation in DR 5-HT axons in the NAc (Dölen et al., 2013). Increases in NAc 5-HT trigger long-term depression (LTD) of excitatory synaptic transmission in NAc spiny neurons *via* activation of presynaptic 5-HT1b receptors (Dölen et al., 2013). Both LTD and social reward are dependent on oxytocin, as genetic deletion of oxytocin receptors in the 5-HT inputs to the NAc abolishes these effects (Dölen et al., 2013). These findings led to the prediction that 5-HT release in the NAc plays a critical role in sociability. Consistent with this prediction, direct optogenetic manipulation of DR 5-HT terminals in the NAc bidirectionally controlled social behavior, which was dependent on 5-HT1b receptors (Walsh et al., 2018). Similarly, recent mechanistic data on (±)3,4-methylenedioxymethamphetamine (MDMA), a drug with strong prosocial effects in humans, further supports the critical function of 5-HT in sociability (Heifets et al., 2019). Infusions of MDMA, which results in large increases in extracellular 5-HT through its actions on the 5-HT transporter (SERT; Green et al., 2003; Hagino et al., 2021), led to significant increases in sociability in the three-chamber task which were blocked by intra-NAc infusions of a 5-HT1b antagonist, but not by an oxytocin receptor antagonist (Heifets et al., 2019). Interestingly, MDMA was also reported to evoke oxytocin release in the NAc, which in turn further modulated 5-HT release to prolong a developmental critical period for social reward learning (Nardou et al., 2019). Considering 5-HT release, unlike DA release, in the NAc is by itself not rewarding (Walsh et al., 2018), the mechanisms by which these two monoamines influence sociability, despite being similarly engaged by oxytocin, must be inherently different. To address this possibility, one recent study used two-color optogenetics to stimulate different glutamatergic inputs to the NAc while 5-HT or DA were bath applied to NAc slices. Recordings from D1 spiny neurons revealed that 5-HT induced LTD in glutamatergic inputs from the basolateral amygdala (BLA), ventral hippocampus, and paraventricular thalamus (PVT), but spared inputs from the medial prefrontal cortex (mPFC; Christoffel et al., 2021). By contrast, DA only induced LTD in PVT inputs. The effects of 5-HT and DA on LTD of different inputs was reproduced with MDMA and methamphetamine, respectively. These discrete physiological effects of 5-HT and DA provide important clues to how they regulate motivated behaviors. Considering these were all presynaptic analyses, we still need data on the postsynaptic effects of 5-HT and DA, although much more is known about postsynaptic DA physiology (Lahiri and Bevan, 2020). Deciphering precisely how DA and 5-HT engage NAc activity to promote social behaviors, and whether an interaction between them is essential, is subject to further investigation.

Outside of mesolimbic circuitries, other corticolimbic brain regions have been implicated in various aspects of sociability. GABAergic neurons in the posterior dorsal medial amygdala (MeApd) dynamically tune social behaviors, with low levels of activity stimulating grooming behaviors and sustained, high frequency activity evoking voracious aggression (Hong et al., 2014). This scalable and tunable activity inspires many hypotheses. For example, the activity-dependent tuning of behavior intensity predicts the release of neuropeptides or neuromodulators from these neurons at high levels of activity. As such, studies have also implicated oxytocin signaling in the MeA in recognition of conspecifics and global deletion of oxytocin resulted in a specific deficit in social memory, which was rescued by infusion of oxytocin into the MeA (Ferguson et al., 2001). A recent study identified GABAergic *Tac1* neurons of the MeA that mediate affiliative touch and consolation behavior directed towards a distressed conspecific *via* projections to the medial preoptic area (Wu et al., 2021).

There is also much evidence for cortical regions in mediating sociability. Activation of the prelimbic cortex (PL) pyramidal neurons significantly reduced time spent in exploring a conspecific in the 3-chamber task and juvenile intruder task (Yizhar et al., 2011). Building on this study it was found that activation of a specific subpopulation of NAc-projecting PL neurons decreased social interaction. Many neurons in the PL-NAc projection were activated during social investigation in a context-dependent manner. Consistent with this, manipulating PL-NAc projections bidirectionally affected interaction time in a social place preference assay, indicating a unique encoding of social and spatial environmental coordinates (Murugan et al., 2017). Similar to the PL, activation of BLA inputs to the NAc also reduced sociability, without affecting palatable food reward (Folkes et al., 2020). Interestingly, activation of this pathway was rewarding, perhaps occluding further reward processing. Facilitating endocannabinoid signaling in the NAc, which led to LTD, buffered the effect of ChR2 stimulation.

Specific subregions of the hippocampus regulate the formation and storage of social memories. Genetically mediated inactivation of a small population of CA2 pyramidal neurons expressing the gene *Amigo2* led to a profound deficit in social memory measured as a deficit in the ability to remember the interaction with a conspecific with no change in sociability *per se* (Hitti and Siegelbaum, 2014). Excitotoxic lesions of the CA2 resulted in deficits in social memory without affecting olfactory memory (Piskorowski et al., 2016). Projections from the supramamillary nucleus (SuM) to the CA2 subdivision of the hippocampus are strongly activated by novel social encounters and activation of the SuM-CA2 pathway can drive exploratory behaviors linked to social novelty (Chen et al., 2020). One recent study identified direct inputs to CA2 *Amigo2* cells from glutamatergic medial septum neurons that are modulated by 5-HT sourced from the median raphe (Wu et al., 2021). Interestingly, social memory was found to require 5-HT-mediated excitation of medial septal inputs to *Amigo2* cells. These studies identify critical circuit nodes of social memory that are largely distinct from those that mediate social approach and reward.

Overall, whether the social circuits that reside outside of canonical mesolimbic pathways also contribute to social reward, reinforcement, and motivation, aside from other features of social interaction dynamics, should be addressed in future work.

## Negative Social Experiences and Drug Intake

A vast body of literature has documented the influence of a variety of negative social experiences (social stressors) on the development of SUDs. In this section, we focus on two major ethologically relevant social stressors: SDS and social isolation stress. Both these stressors increase vulnerability to SUDs. Here we will review the effects of social defeat and social isolation stress on cocaine, alcohol, and morphine dependence highlighting relevant literature spanning the neural circuit and molecular mechanisms by which these stressors enhance vulnerability to drugs.

### SDS and Addiction

There is a complex relationship between mechanisms involved in coping with negative social experiences and those leading to escalated drug intake. The effects of social stress on behavior is a classic inverted U-shaped curve. While mild to moderate forms of stress energize behavior severe and chronic stress paralyzes behavior (Sapolsky, 2015). Clinical studies have shown that for some individuals, moderate alcohol consumption increases prosocial behaviors (de Wit and Sayette, 2018). However, studies have also shown that those who consume alcohol to alleviate social anxiety or in negative social contexts have a greater likelihood of meeting Diagnostic and Statistical Manual (DSM-V) criteria of developing an alcohol use disorder (AUD; Cooper et al., 1992; Sinha, 2001). There is evidence that stress responsivity and coping strategies are good predictors of alcohol use in humans (Brown et al., 1990; Gilpin and Weiner, 2017). SDS is also a potent driver of cocaine use and relapse to cocaine = seeking in those who are dependent on cocaine (Wallace, 1989; Sinha, 2001). Traumatic experiences such as abuse/maltreatment during childhood and domestic violence increase the likelihood of developing opioid use disorder (OUD; Lawson et al., 2013). Opioid abuse can also be perpetuated by continued exposure to traumatic events including physical violence (Cottler et al., 1992), while OUD patients in treatment report higher levels of stress than healthy controls (Hyman et al., 2009).

#### Animal Models of SDS

An important parameter of preclinical models of SDS is the timing of the stress episodes. Repeated exposure to stress is required to produce lasting effects on drug intake in animal models. Ten consecutive days of episodic or intermittent SDS leads to reliable escalations in alcohol consumption in both the continuous access and the intermittent access two-bottle choice models of alcohol consumption (Newman et al., 2012; Norman et al., 2015; Hwa et al., 2016a; Albrechet-Souza et al., 2017). During episodic SDS, an aggressive physical encounter is terminated after a fixed duration or number of attacks. Mice are singly housed between attacks, which occur on consecutive days. In the chronic SDS model, physical confrontations between residents and intruders last 5–10 min, following which residents and intruders are housed next to each other separated by a perforated plexiglass divider. Chronic SDS typically lasts for 10 days (Golden et al., 2011).

Several studies have examined the effects of repeated episodic SDS on relapse to alcohol seeking. In one study, Wistar rats were subjected to five sessions of episodic SDS followed by 9 weeks of social isolation that resulted in a depressive-like state. These rats showed increased motivation to obtain alcohol and increased cue-induced relapse to alcohol seeking, which was reversed by guanfacine, an FDA-approved alpha-adrenergic agonist (Riga et al., 2014). In another study, rats were trained to self-administer alcohol and were subjected to five SDS episodes occurring before the self-administration session after stable responding was achieved. Further, each SDS episode was paired with an odor cue. Exposure to the odor cue paired with SDS led to a modest reinstatement of alcohol self-administration (Funk et al., 2005). This study also found that acute exposure to SDS decreased alcohol self-administration, reduced rats of responding during extinction, and failed to reinstate alcohol seeking.

Chronic SDS in male and female C57BL/6J mice also leads to increased alcohol consumption (Kudryavtseva et al., 1991; Nelson et al., 2018). In the chronic subordinate colony stress (CSC) model, several submissive male mice are housed with a dominant male for days to weeks. Submissive males drink more alcohol in mice, rats, prairie voles, and squirrel monkeys (Blanchard et al., 1987; McKenzie-Quirk and Miczek, 2008; Bahi, 2013; Anacker et al., 2014). Furthermore, exposure to both repeated and chronic SDS leads to profound and long-lasting deficits in sociability (Golden et al., 2011; Newman et al., 2021).

Similar to alcohol, intermittent SDS accelerates the acquisition of cocaine intake. Exposure to a single defeat session leads to enhanced conditioned place preference (CPP) to cocaine (McLaughlin et al., 2006; Montagud-Romero et al., 2015; Tovar-Díaz et al., 2018). Exposure to five consecutive SDS episodes also leads to an enhanced rate of acquisition of cocaine self-administration (Tidey and Miczek, 1997). However, many of these effects of acute SDS on cocaine reward are absent in studies that measure the more protracted effects of stress (Covington and Miczek, 2001; Boyson et al., 2011; Holly et al., 2016). Some studies have shown that episodic SDS enhances the rate of acquisition of cocaine self-administration and increases progressive ratio breakpoints (Covington and Miczek, 2001, 2005; Quadros et al., 2014; Burke and Miczek, 2015). By contrast, prolonged exposure in a chronic SDS model leads to suppression of cocaine intake in both male and female rats and also reduces responding for cocaine in a progressive ratio schedule (Miczek et al., 2011; Shimamoto et al., 2015), illustrating the inverted U curve of stress.

Several studies have examined the effects of SDS on extinction and relapse to cocaine-seeking using a variety of behavioral procedures. As with alcohol, exposure to environmental cues paired with SDS leads to reinstatement of cocaine-seeking (Manvich et al., 2016). The effects of SDS on cocaine reinstatement can be long lasting with studies reporting reinstatement of cocaine seeking after 15 days of abstinence (Covington and Miczek, 2001; Holly et al., 2016). Using the CPP procedure it has been shown that cocaine not only enhances cocaine CPP but this enhanced CPP is more resistant to extinction (Montagud-Romero et al., 2016). These results suggest that exposure to SDS leads to stronger drug-associated memories that are resistant to extinction. Further a single session of SDS reinstated extinguished CPP to cocaine and this required activation of p38a-MAPK activity in the DR (Land et al., 2009; Bruchas et al., 2011).

Although endogenous opioid signaling is profoundly impacted by SDS (Nikulina et al., 2005; Miczek et al., 2008), in preclinical models SDS causes only a transiently increase opioid intake (Badiani et al., 2011). Acute SDS reinstates morphine CPP (Ribeiro Do Couto et al., 2006). Another study indicates that witnessing SDS without physical contact can enhance the reinforcing effects of morphine (Cooper et al., 2017). However, this effect is not long-lasting.

SDS is notoriously difficult to model in females since under most conditions, neither males nor females want to attack a female intruder. However, there have been a few recent studies that have succeeded in modeling SDS in females. In one study, aggressive female CFW (Carworth Farms Webster) mice were housed with castrated males for 3 weeks prior to SDS. Under these conditions, female CFWs were found to aggressively defend their territory and attack any intruder female introduced into their home cage. Using this model, Newman et al. (2019) were able to show that chronic SDS in females enhances alcohol consumption in both the continuous and intermittent access procedures. One recent study used the vicarious SDS model where female C57BL/6J mice vicariously experience the defeat episodes between a CD1 male and a male conspecific for 10 consecutive days. Females that underwent vicarious SDS displayed signs of increased anxiety and depressive like behavior including anhedonia and deficits in social interaction (Iñiguez et al., 2018).

Finally, there have been a handful of studies examining the effects of social stress during adolescence and drug-taking. Adolescent SDS can also significantly escalate drug-seeking and taking (Hoffmann et al., 2000; Tharp-Taylor et al., 2009). Brief episodes of SDS in adolescence were sufficient to increase the breaking point for cocaine in a progressive ration schedule of drug reinforcement and enhance cocaine intake during a 24 h continuous access binge (fixed-ratio schedule of cocaine reinforcement; Burke and Miczek, 2015; Burke et al., 2016). It is unclear if there is a critical period during which adolescents are particularly vulnerable to the effects of social stress. One caveat with the SDS models discussed here is that in most cases, defeated animals are singly housed post stress. Hence, many of the models also have features of social isolation built into them that may be contributing to addiction vulnerability as well.

#### Neural Mechanisms of Social Stress-Induced Escalation of Drug Intake

How does a history of exposure to mild, moderate, or severe social stress impact drug intake? Prolonged exposure to social stress results in neural adaptations that lead to long-term changes in the neural and behavioral response to drugs. Neuromodulators such as 5-HT, DA, and norepinephrine mediate these stress-induced neuroadaptations (Maier and Watkins, 2005; Valentino and Van Bockstaele, 2008; Der-Avakian et al., 2014; Newman et al., 2018b). Dopaminergic mechanisms are particularly intriguing as they are recruited by both SDS and drugs (Di Chiara and Imperato, 1988). A subset of dopamine neurons displays increased burst firing in submissive animals during aggressive encounters with a dominant animal (Anstrom et al., 2009; Barik et al., 2013). Consistent with these findings, SDS also increases extracellular DA levels in the NAc and the PFC (Tidey and Miczek, 1996, 1997; Barik et al., 2013; Han et al., 2015; Holly et al., 2015). Further, the increase in DA was greatest in rats with a prior history of SDS suggesting that repeated exposure to stress can induce neuroplasticity in the mesolimbic DA system.

Repeated SDS also recruits an array of neuropeptide systems, which in turn impact the mesocorticolimbic DA system. These include opioids, glucocorticoids, orexin, oxytocin, CRF, and urocortin (Nikulina et al., 1999; Litvin et al., 2011; Nocjar et al., 2012; Holly et al., 2016). CRF is by far the most well-studied peptide in terms of stress and its impact on the mesolimbic DA system (Koob and Volkow, 2010). Several studies show that CRF can directly modulate DA neurotransmission in the VTA (Ungless et al., 2003; Wanat et al., 2008, 2013) and NAc (Lemos et al., 2012). In a similar manner, the CRF/Urocortin system has been shown to mediate the behavioral effects of several psychostimulants (Lu et al., 2003; Lodge and Grace, 2005) and alcohol (Ryabinin et al., 2002; Valdez et al., 2002; Funk et al., 2007; Zorrilla et al., 2014).

A prevailing theory is that repeated exposure to SDS can sensitize the DA system thereby leading to enhanced neural and behavioral response (Newman et al., 2018b). This mechanism could consequently enhance vulnerability to drug use in individuals exposed to SDS. Studies report higher low dose alcohol-induced DA release in the NAc of stressed animals compared to control animals (Yavich and Tiihonen, 2000) thereby lending support to the cross-sensitization hypothesis (Newman et al., 2018b). Chronic SDS leads to escalated alcohol consumption in susceptible mice that display “depressive-like” symptoms (Nelson et al., 2018). Increased alcohol intake after SDS was negatively correlated with neurokinin-1 receptor and vasopressin expression. Both SDS and drugs activate some of the same brain regions. Repeated SDS activates the bed nucleus of stria terminalis (BNST), and the central amygdala (CeA; Laine et al., 2017). These brain regions also influence drug-seeking. For example, there are CRF neurons in the BNST and CeA that extend long-range projections to the VTA where they influence DA synaptic transmission. Hence, plasticity in these projections could in turn affect DA release in the NAc and modulate drug intake. Systemic or intra-VTA injection of CRF-R1 antagonist CP376395 can reduce alcohol consumption in mice subject to SDS (Hwa et al., 2016a, b; Newman et al., 2018a).

In addition to influencing the reinforcing effects of drugs, SDS has also been shown to potentiate the locomotor stimulating effects of cocaine and d-amphetamine for months after stress (Miczek et al., 1999a, b; Covington and Miczek, 2005; Boyson et al., 2011; Han et al., 2015). Chronic cocaine administration also results in behavioral sensitization, suggesting that drugs and stress may engage similar mechanisms to cause behavioral sensitization to cocaine (Newman et al., 2018b). *In vivo* microdialysis studies indicate that episodic SDS potentiates cocaine and amphetamine-induced DA release in the NAc (Miczek et al., 2011; Holly et al., 2012; Boyson et al., 2014; Han et al., 2015). Stressors may facilitate glutamatergic plasticity in the VTA thereby strengthening associations between drugs of abuse and drug-predictive cues (Robinson and Berridge, 1993; Vanderschuren and Kalivas, 2000). SDS can enhance NMDAR-mediated long-term potentiation (LTP) by increasing IP3R sensitivity in VTA DA neurons (Stelly et al., 2016). Blockade of NMDA receptors in the VTA can prevent social stress-induced escalation of cocaine self-administration (Covington et al., 2008). A single episode of SDS can elicit an increase in extracellular CRF in the VTA. A history of SDS leads to enhanced basal CRF levels in the VTA and also enhanced CRF responses to stress and cocaine administration (Holly et al., 2016; Han et al., 2017). It is possible that elevated CRF tone may interact with mesolimbic DA systems to further enhance stress- or cocaine-induced DA responses. Hence systemic or intra-VTA administration of compounds that antagonize CRF signaling can block SDS escalated cocaine self-administration and relapse to cocaine-seeking (Holly et al., 2016; Han et al., 2017). Studies have also linked CRF and norepinephrine signaling in stress-escalated drug intake. CRF was found to amplify IP3-Ca^2+^ signaling induced by stimulation of α1 adrenergic receptors which in turn promotes NMDAR-mediated glutamatergic plasticity in the VTA. This synergistic mechanism was shown to enhance the learning of cocaine-paired cues (Tovar-Díaz et al., 2018).

In summary, episodic SDS robustly enhances the rewarding and reinforcing effects of cocaine and alcohol. Chronic SDS increases alcohol intake but has a depressive effect on the rewarding and reinforcing effects of stimulants. SDS leads to sensitization of the mesolimbic DA system which leads to escalated drug intake. Future studies should examine the precise cell types that are recruited by both SDS and drugs and neuroadaptations within those cell types using techniques such as activity-dependent cell labeling and single cell transcriptomics.

### Social Isolation Stress and Addiction

Adolescence is a vulnerable time point in human development during which exposure to stress can precipitate psychiatric disorders and drug and alcohol addiction (Heim and Nemeroff, 2001; Chappell et al., 2013; Yorgason et al., 2013; Balogun et al., 2014). In both animal models and human subjects, social isolation stress has been shown to result in profound structural and neurochemical changes in the brain (Jones et al., 2011; Liu et al., 2011). In rodents, depriving social contact during adolescence leads to enduring increases in anxiety-like behavior during adulthood and leads to escalated drug intake. In humans, self-report studies have shown that the number of adverse childhood events is positively correlated with increased prevalence of alcohol and cocaine dependence (Dube et al., 2002; Douglas et al., 2010). Here, we review the animal model of social isolation and the mechanisms by which social isolation impacts drug reward and reinforcement.

#### Animal Model of Social Isolation

A majority of the social isolation studies are carried out during adolescence, a period that is critical for social development. The effects of adolescent social isolation (aSI) on the development of AUD has been an area of intense investigation (Deatherage, 1972; Schenk et al., 1990; Wolffgramm, 1990; Hall et al., 1998). These studies are carried out mostly in rats which are highly social animals living in large groups. Depriving rats of social interaction during adolescence leads to a plethora of behavioral abnormalities in adulthood that are correlated with the development of AUD (Butler et al., 2016). In most studies of aSI, rats are housed in isolation from day 28 onwards for a period of 6-weeks into adulthood. Control rats remained group housed during this period. Rats subject to aSI are hyperactive (Chappell et al., 2013; Butler et al., 2014a; Ishikawa et al., 2014), and exhibit deficits in sensory motor gating immediately post isolation and well into adulthood (McCool and Chappell, 2009; Liu et al., 2011; Ko and Liu, 2015). aSI rats display increased anxiety-like behavior on the elevated plus maze (Hall et al., 1998; McCool and Chappell, 2009; Chappell et al., 2013). While aSI rats do not differ in the acquisition of fear conditioning when compared to control rats, they do exhibit a significant reduction in fear extinction (Skelly et al., 2015). aSI also resulted in depressive like behaviors in rats (Brenes et al., 2008) with studies revealing reduced swim time and increased immobility on the forced swim test which was reduced by treatment with the antidepressant, Desipramine (Simpson and Kelly, 2012).

Since aSI alters a wide variety of behaviors associated with increased vulnerability to AUD, several groups have tested the effects of aSI on alcohol intake. These studies report increased alcohol intake after aSI (Deatherage, 1972; Schenk et al., 1990; Wolffgramm, 1990; Hall et al., 1998). aSI enhances alcohol consumption in both the continuous and intermittent access procedures in outbred rats (McCool and Chappell, 2009; Chappell et al., 2013). The effects of aSI are long-lasting and endure for up to 8 weeks after isolation (Chappell et al., 2013; Skelly et al., 2015). This increase in alcohol consumption observed in aSI rats was attenuated by systemic injection of the KOR antagonist norBNI (Chappell et al., 2013; Skelly et al., 2015), implicating the dynorphin/KOR system in mediating the effects of aSI on alcohol drinking.

aSI also enhances operant responding for alcohol in rats. Using an operant procedure that discriminates between appetitive and consummatory aspects of alcohol consumption, it was shown that aSI enhances both these aspects of alcohol consumption. No changes were observed in operant sucrose consumption between the stressed and control rats (McCool and Chappell, 2009). aSI also enhanced CPP for alcohol and amphetamine in rats (Whitaker et al., 2013). This study found that amphetamine-associated contextual memories were also resistant to extinction in aSI rats. Interestingly, multiple studies have reported that females are resilient to the effects of aSI. No changes in anxiety-like behaviors were observed in female rats after aSI, but transiently increased alcohol consumption was found (Butler et al., 2014b). The neural underpinnings of this sex difference in the effects of aSI on anxiety-like behavior remain known. The effects of aSI also seem to be species-specific with mice being resilient to the effects of aSI on alcohol consumption. One recent study found that aSI in C57BL/6J mice resulted in a hypersocial phenotype in males and females and an anxiolytic phenotype in females. Alcohol consumption was not affected in either of the sexes (Rivera-Irizarry et al., 2020).

In addition to alcohol, aSI also enhances the behavioral response to other drugs including cocaine and morphine in rats. aSI enhances CPP to higher doses of cocaine (Zakharova et al., 2009; Grotewold et al., 2014). In males, aSI also enhances self-administration of lower doses of cocaine that group housed animals do not self-administer suggesting aSI may increase sensitivity to cocaine (Schenk et al., 1987; Howes et al., 2000; Gipson et al., 2011; Smith et al., 2014). aSI also increases the amount of cocaine self-administered (Schenk et al., 1987; Boyle et al., 1991) and increases break point for cocaine in a progressive ratio procedure in male rats and this effect persists even after resocialization (Baarendse et al., 2014). Finally, aSI enhances cue-induced reinstatement to cocaine in both male and female mice (Fosnocht et al., 2019). In contrast to cocaine and ethanol, aSI decreases low-dose morphine CPP in males (Wongwitdecha and Marsden, 1996) suggesting that the effects of aSI on CPP are drug-specific. By contrast, aSI enhances morphine self-administration in rats (Marks-Kaufman and Lewis, 1984).

Social isolation in adult mice results in a plethora of behavioral abnormalities. In one study chronic (28 days) isolation of adult (9-week old) male C57BL/6J mice resulted in increased anxiety-like behavior as well as depressive-like behavior as measured by the forced swim and tail suspension tests. These behaviors were accompanied by molecular changes including lower mRNA levels of the BDNF-7 splice variant and immediate early genes including Arc, Egr1, and C-Fos in the hippocampus and prefrontal cortex of stressed mice (Ieraci et al., 2016). Multiple studies have reported that chronic (2 weeks) of social isolation in adult mice also results in enhanced aggression and threat responsivity (Zelikowsky et al., 2018a). One study found that chronic social isolation in adults leads to increased aggression towards a submissive intruder, increased reactivity to a footshock stimulus, increased tail rattling, persistent freezing to a looming disk, and reduced social interaction (Zelikowsky et al., 2018b). This study also found a striking increase in expression of the neuropeptide Tachykinin 2 (Tac2) in brain regions involved in emotional and social behaviors including the BNST and the CeA and went on to demonstrate a critical role for Tac2 and its cognate receptor Neurokinin 3 receptor (NK3R) in the behavioral responses to chronic social isolation. Chronic social isolation in adult rats also resulted in increased anhedonia- like behavior which was mediated by increased CREB activation and altered CREB-dependent transcription in the NAc shell subdivision (Wallace et al., 2009). In contrast with aSI models, the effects of chronic social isolation in adults on addictive behaviors is modest and variable. One recent study examined the effects of social isolation in adult mice on ethanol intake and found that social isolation increased alcohol intake and preference which were mediated by microglia activation and alterations in serotonin levels in the DR (Lee et al., 2021).

#### Neural Mechanisms of aSI-Induced Escalation of Drug Intake

Investigations into the neural and molecular mechanisms of the behavioral abnormalities associated with aSI have once again focused mostly on the mesolimbic DA system (Butler et al., 2016). *In vivo* electrophysiological studies revealed that aSI increased the firing rate and burst-like activity of DA neurons in the VTA (Fabricius et al., 2010). aSI was also shown to increase LTP of NMDA-receptor mediated glutamatergic transmission in putative VTA DA neurons (Whitaker et al., 2013). Fast scan cyclic voltammetry studies have revealed significant increases in both electrically stimulated DA release and the rate of DA reuptake in accumbal slices from aSI rats and these changes persisted into adulthood (Yorgason et al., 2016). aSI also increases DA transporter (DAT) expression in the ventral and dorsal striatum in aSI rats. One study using microdialysis revealed that aSI was associated with reduced baseline DA levels and pretreatment with a KOR antagonist norBNI increased DA levels selectively in aSI rats suggesting increased functional responsiveness of KORs in the NAc after aSI (Karkhanis et al., 2016). aSI rats also shower greater accumbal DA and norepinephrine release in response to an acute ethanol injection (Karkhanis et al., 2014). The BLA plays a major role in regulating anxiety-like behaviors. aSI led to increased DAT expression and consequently reduced baseline DA levels in the BLA. Further acute ethanol injections led to enhanced DA levels in the BLA in aSI rats compared to group housed controls (Karkhanis et al., 2015). aSI also increased the intrinsic excitability of BLA pyramidal neurons. Significant deficits were also observed in slow and medium afterhyperpolarizations, suggesting lower thresholds for burst firing. Biochemical studies revealed reduced expression of small conductance potassium channels SK2 and SK3. Allosteric potentiation of SK channels with 1-EBI0 restored intrinsic excitability and reversed aSI induced increases in anxiety-like behaviors (Rau et al., 2015).

In summary, these results provide strong evidence that aSI enhances drug-seeking and taking in adults and these effects are mostly associated with changes in the mesolimbic DA system. However, there seems to be a marked species difference in the effects of aSI on drug intake with most of the literature focusing on studies in rats. The mechanisms underlying the species-specific nature of the effects of aSI on drug intake are not known. While social isolation in adults results in a plethora of behavioral effects including anxiety, aggression, and depressive like behavior, its effects on drug intake are modest and variable. Hence, it appears that there is a critical period during development when social isolation increases vulnerability to addiction.

## The Interplay of Drug Use and Social Behavior

While negative social experiences powerfully influence the development and maintenance of addictive behaviors, a history of drug use can also affect social behaviors, closing the loop on a continuum of socio-addictive behaviors that co-influence each other. Alterations in sociability are emerging as key determinants of drug-seeking and a major factor in the negative affective states that drive relapse (in addition to many co-morbid psychiatric conditions; Strang et al., 2020; Venniro et al., 2020b; Welsch et al., 2020). This section will discuss some of the relevant data describing deficits in social drive resulting from a history of drug use and propose models for how social deficits are risk factors for drug relapse.

### Effects of Drug History on Social Behavior

In general, substance use disorders exert negative influences over social behavior, such that individuals exhibit social avoidance during drug withdrawal and choose to engage in drug-taking over naturally rewarding social interactions. Reductions in social behavior promote individuals to consume drugs more often and in larger amounts, leading to greater social avoidance and isolation, resulting in an escalating cycle of maladaptive drug-seeking and social avoidance that promote each other (Volkow et al., 2011). This is particularly apparent when thinking about the consequences of social isolation during the COVID-19 pandemic, which has likely contributed to the massive increase in opioid overdoses (>100,000 in 2020, up from ~47,000 in 2017; Kosten and Petrakis, 2021). As OUD is a chronically relapsing, iatrogenic SUD, it is imperative to identify novel and effective therapeutic approaches, which may require social components. Naloxone-precipitated morphine withdrawal in morphine-dependent mice reduced preference for a social context in the 3-chamber sociability task (Valentinova et al., 2019). This change in social behavior depended on reduced excitatory synaptic drive in DR-projecting lateral habenula neurons that was mediated by tumor necrosis factor-alpha activity (Valentinova et al., 2019). This is not all too surprising, however, due to the fact that precipitated opioid withdrawal induces physical sickness and somatic signs (jumping, tremors, fever, etc.), consistent with cytokine signaling. Therefore, it is possible the reduced social preference in this study resulted from general sickness or malaise, rather than being specific to dysfunctions in social circuitry. While animals and humans alike will appear asocial during acute opioid withdrawal, this effect is more likely an epiphenomenon related to generalized illness and not a pure modulation of emotional state.

Contrary to acute opioid withdrawal, several weeks of abstinence, known as protracted opioid withdrawal, generally leads to a strong incubation of craving (Pickens et al., 2011) and disrupts basic social behavior (free dyadic interactions; Goeldner et al., 2011). Interestingly, the effects on social behavior were not present after only 1 week of withdrawal, suggesting that it is a slow and long-term adaptation to the absence of morphine. This study also identified impairments of 5-HT function in the DR and found that chronic fluoxetine was sufficient to restore normal social behavior during protracted withdrawal (Goeldner et al., 2011). In a separate article, this same group showed that 5-HT1A receptor function was enhanced in the PFC but decreased in the DR during protracted morphine withdrawal, further supporting a role for dysfunctional 5-HT systems after a history of drug use that may mediate social deficits (Lutz et al., 2011). Along similar lines, the 5-HT2C receptor agonist Lorcaserin attenuated behavioral sensitization to heroin, including changes in locomotor behavior and immobility in the forced swim test (Wu et al., 2015). This role for KORs was replicated in a separate study where mice were administered the long-lasting KOR antagonist norBNI after escalating doses of heroin (Lalanne et al., 2017). Interestingly, norBNI was shown to prevent social deficits in heroin-dependent mice in protracted withdrawal whether it was given 24 h after or several weeks after the last heroin injection (Lalanne et al., 2017). These results indicate that chronic blockade of KORs (systemically) can prevent and reverse the social consequences of protracted opioid withdrawal—strong implications for therapeutic strategies targeting KORs.

The effects on KORs are fascinating in light of the previously discussed neuromodulatory mechanisms of prosocial behavior. Since 5-HT and DA are critical drivers of social behaviors, it is intriguing to consider that KORs are known to reduce both extracellular levels of 5-HT and DA in the NAc (Spanagel et al., 1992; Schindler et al., 2012; Pirino et al., 2020; Tao et al., 2020). The studies discussed above present data with systemic or global inhibition of KOR function. Whether the KOR mechanism that underlies opioid withdrawal social deficits is specific to certain brain regions or neurochemicals, such as 5-HT or DA, should be the focus of future investigations.

Another study also identified deficits in social preference in the 3-chamber task after prolonged morphine abstinence that was reversed by administration of the oxytocin analog carbetocin (Zanos et al., 2014). Since this agent was given just prior to sociability testing, the positive result is not too surprising considering that oxytocin signaling is sufficient to enhance social behaviors on its own. The same group subsequently found that morphine abstinence-induced social deficits were associated with upregulations of mGluR5 across the brain (Zanos et al., 2016), although a direct contribution of mGluR5 to social deficits was not determined.

Reductions in social behavior have also been found in animal subjects undergoing withdrawal from chronic alcohol (ethanol) drinking. After repeated withdrawal episodes from a forced ethanol diet, rats exhibited large decreases in social interaction that were prevented by administration of a 5-HT2C receptor antagonist and a 5-HT1A receptor partial agonist (Overstreet et al., 2003). This same group subsequently found that ethanol withdrawal-induced social deficits were counteracted by injection of 5-HT2C receptor inverse agonist into the amygdala or injection of the same 5-HT1A partial agonist into the DR (Overstreet et al., 2003). Chronic intermittent ethanol vapor exposure that renders animals ethanol-dependent, leads to strong social interaction deficits in withdrawal that are blocked by the injection of an arginine vasopressin receptor antagonist, but not an oxytocin antagonist, into the central amygdala (Harper et al., 2019). Adolescent rats have also been observed to display social deficits after chronic ethanol treatment, which depend on activity in CRF and 5-HT systems (Wills et al., 2009). Further supporting a role for altered 5-HT function during ethanol withdrawal, acute and protracted ethanol withdrawal produced deficits in social approach behavior that were reversed by the 5-HT1A agonist buspirone (Lowery-Gionta et al., 2015). Similar social approach changes were identified to depend on 5-HT2C receptors, and these were localized to the ventral BNST where they increase the excitability of an unknown population of neurons (Marcinkiewcz et al., 2015). One interesting study transplanted microbiota from humans with AUD into mice and identified a significant social deficit (Leclercq et al., 2020). It was also found that the changes in social behavior were mediated by reduced hepatic synthesis of beta-hydroxybutyrate, since a ketogenic diet normalized both the behavioral and metabolic effects (Leclercq et al., 2020). Altogether, it appears that a history of ethanol drinking and ethanol withdrawal robustly decrease social drive. This behavioral change is mediated by many factors, but modifications in 5-HT signaling may serve as a common mechanism.

It appears that changes in social behavior during drug withdrawal are specific to depressant drugs (i.e., opioids, alcohol). This is evidenced by work showing that withdrawal to chronic amphetamine or nicotine exposure leads to anhedonia-like responses but not changes in social behavior (Irvine et al., 2001; Morley et al., 2001; Der-Avakian et al., 2014). By contrast, withdrawal to chronic cocaine appears to promote social deficits 7 days into withdrawal but by 14 days the effect is gone, mediated by CRF2 receptors (Morisot et al., 2018). However, one article reports mild reductions in social preference 18 h after a single injection of amphetamine, which was reversed by diazepam (Rincón-Cortés et al., 2018). While the effects of depressant drugs are more robust when examining consequences on social behavior, stimulant drugs may have a more complex relationship with the long-term outcomes. A comprehensive comparison of different drug class effects on social changes during self-administration and withdrawal is needed.

### Social vs. Drug Reward Choice

If it is true that changes in social behaviors are predictive of inflexible drug use, drug-seeking, and relapse, then behavioral assays that evaluate the choice to engage with a drug stimulus over a social stimulus are essential. This is similar to the notion that during late stages of addiction and drug dependence, drug rewards outcompete natural rewards and lay a foundation for rigid motivational states that guide users towards the drug of choice and away from other reinforcers. This is a cardinal feature of human addiction that is targeted by contingency management and community-reinforcement therapies (Venniro et al., 2020b). As social stimuli are one of the most complex and immersive forms of natural reward, not only in our modern world but also across the animal kingdom, understanding the dynamics between the motivation to engage with a social stimulus over a desired drug may reveal important aspects of the addiction process. Indeed, this has been evaluated for cocaine where rats switch their preference for a cocaine context to a social context after a series of conditioning sessions (Fritz et al., 2011; Zernig et al., 2013). This was paralleled by a reversal of brain activity patterns that was predicted to bias approach to a social environment over a cocaine one. Similar findings were found in cocaine self-administration tasks where group-housed rats self-administered less cocaine if their cagemates were cocaine naïve, perhaps serving as a “social buffering” effect (Smith, 2012). While these data support the idea that competing neuronal substrates mediate social vs. drug reward, it is unclear what the mechanisms are and whether there are individual differences in these effects (i.e., some subjects that are resistant to social engagement with different brain activity profiles).

Venniro et al. (2018, 2019, 2020b) have recently produced a series of elegant and influential articles demonstrating that social reward indeed competes with drug reward and animal subjects often choose a social reinforcer over a drug reinforcer. In this procedure, subjects are trained to self-administer a drug reward and access to a conspecific in separate sessions, and then are given the choice in a test session (Venniro et al., 2018, 2020a). Remarkably, most subjects vigorously self-administered a social stimulus over a drug, and this social choice was present in rats that exhibited high levels of addictive behaviors including high progressive ratio breakpoints, compulsive reward taking when challenged with footshocks, and incubation of craving after prolonged abstinence. Despite these high levels of motivation for drugs, a social stimulus still outcompeted drug-taking and seeking. A follow up study examined whether social-choice-induced abstinence (as opposed to forced abstinence) could affect time-dependent increases in heroin seeking (Venniro et al., 2019) This study found that social choice attenuated lever pressing for heroin after 15 days of abstinence (Venniro et al., 2019). A follow up study examining methamphetamine seeking identified increased activity of central amygdala PKCdelta neurons in rats given the choice to socialize (Venniro et al., 2020a). One interesting caveat of this model is the time-dependence of the social reward. Unlike rats, where a vast majority if not 100% of rats choose a social stimulus over a drug, a proportion of human drug users will continue to use drugs despite the availability of social support and avoid social contact (Heyman, 2013). In the choice model, there is no delay between the operant response and access to the social stimulus. In humans, however, social reward is often delayed. When a 60 s delay is introduced into the choice task, rats ended up choosing the social stimulus as often as the heroin stimulus, reproducing the variability seen in humans. This concept was tested for cocaine self-administration and the delay was found to reduce preference for social access (Venniro et al., 2021). In addition, increasing the response requirements (increasing fixed ratio for each social reward) reduced social preference and enhanced cocaine preference, with notable inter-individual variability. Therefore, the dynamics surrounding the interplay of social motivation and drug motivation may be key to predicting relapse rates and using social interventions to break persistent addiction cycling.

These remarkable data indicate that there are other neurological mechanisms engaged under certain circumstances to shift the motivation to obtain the drug to that aimed towards socializing. Questions do remain on at least two dimensions: (1) what are the individual differences in animals that do not choose social stimuli over drugs and is there a unique neurobiology in these individuals; and (2) what are the effects on basic social interaction and approach behaviors after extended drug self-administration? Future studies should focus on disentangling the bidirectional influence of social behavior on drug-seeking/taking in different individuals to identify potential differences in the sensitivity to this profound intervention. If differences do emerge, they may provide clues on how to effectively implement social strategies to curb drug-seeking behaviors in treatment-resistant humans.

### Interactions of Social and Reward Circuits

Do reward and reinforcement from social activities emerge from inherently different mechanisms than those generated by drugs of abuse? Two distinct types of reward likely require differences in underlying neural circuitry. Natural vs. drug reward have been distinguished in countless studies, however generally using palatable stimuli such as sucrose as a substitute for the drug of interest. Only recently has social reward been adopted as a competing reward with addictive substances, despite its critical relevance to the pathogenesis of substance use disorders.

A fascinating pharmacological tool may have the potential to clarify this question. As mentioned above, MDMA is an amphetamine derivative with remarkable properties. Unlike amphetamine or methamphetamine, MDMA’s primary mechanism of action is through evoking reverse transport of SERT (as opposed to DAT). This non-vesicular release *via* reverse transport produces supraphysiological levels of 5-HT, which mediate MDMA’s prosocial effects (Heifets et al., 2019). MDMA also performs a similar action on the dopamine transporter (DAT), but with less affinity. In contrast, amphetamine and methamphetamine also trigger reverse transport of both modulators but with a much larger affinity for DAT than for SERT. This has led to the hypothesis that most amphetamines have high abuse liability while MDMA maintains a low abuse liability due to differential effects on DA and 5-HT. Extrapolating to other drugs of abuse, all of which drive DA release, but not so much 5-HT release, a pattern is emerging where the degree of 5-HT engagement may dictate the degree of addictive behavior. In support of this notion, a recent article showed that 5-HT signaling in the striatum attenuates compulsive cocaine-seeking through suppression of orbitofrontal inputs *via* 5-HT1B receptors (Li et al., 2021). Since cocaine inhibits SERT in addition to DAT, a mouse line with a mutated SERT that cocaine cannot bind to was observed to exhibit much larger degrees of compulsive cocaine seeking. This implies that 5-HT has a dampening effect on drug-seeking. This idea is supported by a study with SERT knockout rats that were shown to self-administer MDMA much more readily and at larger amounts (Oakly et al., 2014).

The simplest explanation for these profound effects is that 5-HT competes with DA somewhere in the brain. Data showing that 5-HT decreases upon reward consumption support this proposition. In addition, considering the inverse relationship drug-taking/seeking has with sociability, it is tempting to hypothesize that as addiction cycles progress, 5-HT function becomes increasingly dampened while the DA function is increasingly engaged by drug cues. Furthermore, as decreased 5-HT signaling promotes social avoidance, unopposed DA signaling can easily mediate the drive to relapse in the face of drug cues. It can also promote the effects of stress on relapse by setting the stage for a negative affective state that motivates an individual to self-medicate. The 5-HT deficit may have a two-pronged effect on substance use disorders by: (1) evoking states of social isolation; and (2) leaving vulnerable reward circuitry unopposed. Therefore, relapse may be precipitated at both behavioral and physiological levels. Exploiting social circuitry to attenuate drug-seeking and relapse may prove useful in treating severe substance use disorders. As such, therapeutic approaches that aim to boost the function of social circuits and enhance the rewarding effects of social interaction are highly promising, not only for substance use disorders but many other psychiatric conditions.

## Protective Effects of Prosocial Behaviors

The notion that negative social experiences and reduced social drive have a promotional effect on drug-taking and seeking is well supported by preclinical and clinical literature. This relationship extends to the prediction that positive prosocial interactions and experiences have protective effects over drug-taking, drug-seeking, and SUDs in general. While most of the literature to date has focused on harmful manipulations (i.e., negative changes to social environments) and inferred beneficial effects from controls (with presumably “normal” or non-destructive social conditions), studies that manipulate social conditions in the positive direction support a protective role for positive prosocial behavior. This is most readily exemplified by the so-called “rat park” experiments where rat subjects were group housed in large cages with significant social and non-social enrichment (Alexander et al., 1978, 1981; Hadaway et al., 1979). These landmark articles all showed that enriched housing conditions reduce opioid consumption. In addition, one study (Alexander et al., 1981) showed that previously isolated rats that were subsequently housed in enriched conditions drank less than purely isolated rats, suggesting that enriching social conditions can attenuate intake. We direct the readers to this short review for further analysis of rat park studies (Gage and Sumnall, 2019). More recent studies have identified protective roles for social behavior and by extension oxytocin in reducing drug and alcohol intake (reviewed in Tops et al., 2014 and McGregor and Bowen, 2012). In a study on adolescents in Thailand, community disorganization was strongly associated with self-reported substance use while prosocial activities served as a protective factor (Wongtongkam et al., 2015). Enhancing sociability, through environmental conditions or neuromodulation, has a clear protective effect on drug-taking. Whether these effects are also due to stress-buffering or attenuation of other symptoms (that sociability can influence) remain unknown. Regardless, therapeutic approaches that capitalize on these effects have promise in breaking addiction cycles.

## Outlook and Future Challenges

**Figure 1:**
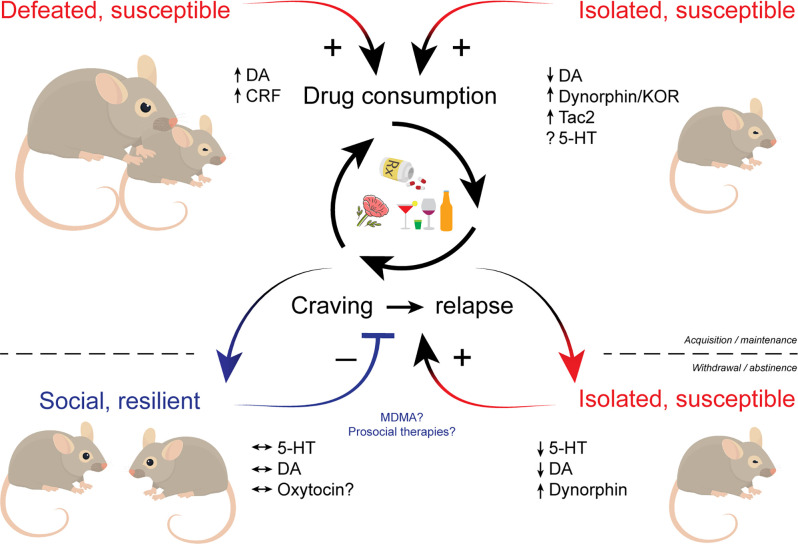
Schematic illustrating the influence of social factors across phases of addiction. Social defeat and social isolation stress facilitate the acquisition and promote the maintenance of drug use. Drug withdrawal and prolonged abstinence promote social isolation that in turn increases susceptibility to drugs. Individuals who maintain proper social drive may be resilient to drug cravings and relapse. Multiple neuromodulators including dopamine, serotonin, and various neuropeptides play key roles in social influences over addictive behaviors. Therapeutic approaches that bolster sociability like MDMA may hold promise in treating SUDs. MDMA, (±)3,4-methylenedioxymethamphetamine; SUDs, substance use disorders.

The relationship between social experiences and addiction is bidirectional. Negative social experiences such as those experienced during aversive social interactions or during withdrawal from chronic drug use have the potential to powerfully influence drug-taking and seeking. By contrast, positive and prosocial interactions can buffer some of these negative affective states and aid in addiction recovery. While it is clear that many distributed brain circuits are involved in different features of sociability, the critical roles of striatal DA and 5-HT may be particularly compromised over the course of the addiction process (Figure 1). Based on the available data, a convergence point between drug-seeking behavior and social behavior may be represented in the mechanisms these modulators orchestrate in the NAc. It is also noted that many of the other structures known to shape social dynamics have also been shown to be modified by repeated drug exposure. All of these fascinating regions are subject to further investigation. Not only does understanding the relationship social and addictive behaviors have with one another provide insight into how these individual behaviors are generated, it also brings into focus the obvious importance of treatment approaches focusing on social factors. In humans, most rewards that compete with drugs are social in nature. This is already being addressed with the increasing use of animal models that incorporate social factors and the continued implementation of social-based treatments for addiction, such as the community reinforcement approach since the 1970s. This approach is a psychosocial intervention that aims to help individuals with SUDs rearrange their lifestyles so that healthy, drug-free living becomes rewarding and competes with drug use. Patients are encouraged to become progressively involved in alternative non-substance-related pleasant social activities, and to work on enhancing enjoyment they receive within the community of their family and job (Meyers et al., 2011). We are hopeful that more data from basic neuroscience examinations of these behavioral relationships will inspire more creative and effective interventions for individuals with SUDs.

## Author Contributions

MP and RM contributed equally to the manuscript. FP contributed to the social isolation portion. All authors contributed to the article and approved the submitted version.

## Conflict of Interest

The authors declare that the research was conducted in the absence of any commercial or financial relationships that could be construed as a potential conflict of interest.

## Publisher’s Note

All claims expressed in this article are solely those of the authors and do not necessarily represent those of their affiliated organizations, or those of the publisher, the editors and the reviewers. Any product that may be evaluated in this article, or claim that may be made by its manufacturer, is not guaranteed or endorsed by the publisher.

## References

[B1] Abu-Akel A. Reniers R. L. Wood S. J. (2016). Visual-spatial processing and working-memory load as a function of negative and positive psychotic-like experiences. Cogn. Neuropsychiatry 21, 402–411. 10.1080/13546805.2016.1206873 27438900

[B2] Albrechet-Souza L. Viola T. W. Grassi-Oliveira R. Miczek K. A. de Almeida R. M. M. (2017). Corticotropin releasing factor in the bed nucleus of the stria terminalis in socially defeated and non-stressed mice with a history of chronic alcohol intake. Front. Pharmacol. 8:762. 10.3389/fphar.2017.00762 29118713 PMC5660971

[B3] Alexander B. K. Beyerstein B. L. Hadaway P. F. Coambs R. B. (1981). Effect of early and later colony housing on oral ingestion of morphine in rats. Pharmacol. Biochem. Behav. 15, 571–576. 10.1016/0091-3057(81)90211-2 7291261

[B4] Alexander B. K. Coambs R. B. Hadaway P. F. (1978). The effect of housing and gender on morphine self-administration in rats. Psychopharmacology (Berl) 58, 175–179. 10.1007/BF00426903 98787

[B5] Aloise-Young P. A. Kaeppner C. J. (2005). Sociometric status as a predictor of onset and progression in adolescent cigarette smoking. Nicotine Tob. Res. 7, 199–206. 10.1080/14622200500055277 16036276

[B6] Anacker A. M. Smith M. L. Ryabinin A. E. (2014). Establishment of stable dominance interactions in prairie vole peers: relationships with alcohol drinking and activation of the paraventricular nucleus of the hypothalamus. Soc. Neurosci. 9, 484–494. 10.1080/17470919.2014.931885 24963825 PMC4349411

[B7] Anstrom K. K. Miczek K. A. Budygin E. A. (2009). Increased phasic dopamine signaling in the mesolimbic pathway during social defeat in rats. Neuroscience 161, 3–12. 10.1016/j.neuroscience.2009.03.023 19298844 PMC4445890

[B8] Baarendse P. J. Limpens J. H. Vanderschuren L. J. (2014). Disrupted social development enhances the motivation for cocaine in rats. Psychopharmacology (Berl) 231, 1695–1704. 10.1007/s00213-013-3362-8 24311358 PMC3969396

[B9] Back S. Dansky B. S. Coffey S. F. Saladin M. E. Sonne S. Brady K. T. (2000). Cocaine dependence with and without post-traumatic stress disorder: a comparison of substance use, trauma history and psychiatric comorbidity. Am. J. Addict. 9, 51–62. 10.1080/10550490050172227 10914293

[B10] Badiani A. Belin D. Epstein D. Calu D. Shaham Y. (2011). Opiate versus psychostimulant addiction: the differences do matter. Nat. Rev. Neurosci. 12, 685–700. 10.1038/nrn3104 21971065 PMC3721140

[B11] Bahi A. (2013). Increased anxiety, voluntary alcohol consumption and ethanol-induced place preference in mice following chronic psychosocial stress. Stress 16, 441–451. 10.3109/10253890.2012.754419 23194312

[B12] Balogun O. Koyanagi A. Stickley A. Gilmour S. Shibuya K. (2014). Alcohol consumption and psychological distress in adolescents: a multi-country study. J. Adolesc. Health 54, 228–234. 10.1016/j.jadohealth.2013.07.034 24064281

[B13] Barik J. Marti F. Morel C. Fernandez S. P. Lanteri C. Godeheu G.. (2013). Chronic stress triggers social aversion *via* glucocorticoid receptor in dopaminoceptive neurons. Science 339, 332–335. 10.1126/science.1226767 23329050

[B14] Barnes G. M. Farrell M. P. (1992). Parental support and control as predictors of adolescent drinking, delinquency and related problem behaviors. J. Marriage Family 54, 763–776. 10.2307/353159

[B15] Barnes G. M. Reifman A. S. Farrell M. P. Dintcheff B. A. (2000). The effects of parenting on the development of adolescent alcohol misuse: a Six-Wave latent growth model. J. Marriage Family 62, 175–186. 10.1111/j.1741-3737.2000.00175.x

[B16] Beier K. T. Steinberg E. E. DeLoach K. E. Xie S. Miyamichi K. Schwarz L.. (2015). Circuit architecture of VTA dopamine neurons revealed by systematic input-output mapping. Cell 162, 622–634. 10.1016/j.cell.2015.07.015 26232228 PMC4522312

[B17] Blanchard R. J. Hori K. Tom P. Blanchard D. C. (1987). Social structure and ethanol consumption in the laboratory rat. Pharmacol. Biochem. Behav. 28, 437–442. 10.1016/0091-3057(87)90502-8 3432310

[B18] Boyle A. E. Gill K. Smith B. R. Amit Z. (1991). Differential effects of an early housing manipulation on cocaine-induced activity and self-administration in laboratory rats. Pharmacol. Biochem. Behav. 39, 269–274. 10.1016/0091-3057(91)90178-5 1946568

[B19] Boyson C. O. Holly E. N. Shimamoto A. Albrechet-Souza L. Weiner L. A. DeBold J. F.. (2014). Social stress and CRF-dopamine interactions in the VTA: role in long-term escalation of cocaine self-administration. J. Neurosci. 34, 6659–6667. 10.1523/JNEUROSCI.3942-13.2014 24806691 PMC4012317

[B20] Boyson C. O. Miguel T. T. Quadros I. M. Debold J. F. Miczek K. A. (2011). Prevention of social stress-escalated cocaine self-administration by CRF-R1 antagonist in the rat VTA. Psychopharmacology (Berl) 218, 257–269. 10.1007/s00213-011-2266-8 21468623 PMC3166547

[B21] Brenes J. C. Rodríguez O. Fornaguera J. (2008). Differential effect of environment enrichment and social isolation on depressive-like behavior, spontaneous activity and serotonin and norepinephrine concentration in prefrontal cortex and ventral striatum. Pharmacol. Biochem. Behav. 89, 85–93. 10.1016/j.pbb.2007.11.004 18096212

[B22] Brown S. A. Vik P. W. McQuaid J. R. Patterson T. L. Irwin M. R. Grant I. (1990). Severity of psychosocial stress and outcome of alcoholism treatment. J. Abnorm. Psychol. 99, 344–348. 10.1037//0021-843x.99.4.344 2266207

[B23] Bruchas M. R. Schindler A. G. Shankar H. Messinger D. I. Miyatake M. Land B. B.. (2011). Selective p38α MAPK deletion in serotonergic neurons produces stress resilience in models of depression and addiction. Neuron 71, 498–511. 10.1016/j.neuron.2011.06.011 21835346 PMC3155685

[B24] Buffington S. A. Di Prisco G. V. Auchtung T. A. Ajami N. J. Petrosino J. F. Costa-Mattioli M. (2016). Microbial reconstitution reverses maternal diet-induced social and synaptic deficits in offspring. Cell 165, 1762–1775. 10.1016/j.cell.2016.06.001 27315483 PMC5102250

[B25] Burke A. R. DeBold J. F. Miczek K. A. (2016). CRF type 1 receptor antagonism in ventral tegmental area of adolescent rats during social defeat: prevention of escalated cocaine self-administration in adulthood and behavioral adaptations during adolescence. Psychopharmacology (Berl) 233, 2727–2736. 10.1007/s00213-016-4336-4 27251131 PMC4919183

[B26] Burke A. R. Miczek K. A. (2015). Escalation of cocaine self-administration in adulthood after social defeat of adolescent rats: role of social experience and adaptive coping behavior. Psychopharmacology (Berl) 232, 3067–3079. 10.1007/s00213-015-3947-5 25943168 PMC4515153

[B27] Butler T. R. Ariwodola O. J. Weiner J. L. (2014a). The impact of social isolation on HPA axis function, anxiety-like behaviors and ethanol drinking. Front. Integr. Neurosci. 7:102. 10.3389/fnint.2013.00102 24427122 PMC3877772

[B28] Butler T. R. Carter E. Weiner J. L. (2014b). Adolescent social isolation does not lead to persistent increases in anxiety-like behavior or ethanol intake in female long-evans rats. Alcohol. Clin. Exp. Res. 38, 2199–2207. 10.1111/acer.12476 25092210 PMC4146661

[B29] Butler T. R. Karkhanis A. N. Jones S. R. Weiner J. L. (2016). Adolescent social isolation as a model of heightened vulnerability to comorbid alcoholism and anxiety disorders. Alcohol. Clin. Exp. Res. 40, 1202–1214. 10.1111/acer.13075 27154240 PMC5131257

[B30] Chappell A. M. Carter E. McCool B. A. Weiner J. L. (2013). Adolescent rearing conditions influence the relationship between initial anxiety-like behavior and ethanol drinking in male Long Evans rats. Alcohol. Clin. Exp. Res. 37, E394–E403. 10.1111/j.1530-0277.2012.01926.x 22924742 PMC3529208

[B31] Chen S. He L. Huang A. J. Y. Boehringer R. Robert V. Wintzer M. E.. (2020). A hypothalamic novelty signal modulates hippocampal memory. Nature 586, 270–274. 10.1038/s41586-020-2771-1 32999460

[B32] Chevallier C. Kohls G. Troiani V. Brodkin E. S. Schultz R. T. (2012). The social motivation theory of autism. Trends Cogn. Sci. 16, 231–239. 10.1016/j.tics.2012.02.007 22425667 PMC3329932

[B33] Christoffel D. J. Walsh J. J. Hoerbelt P. Heifets B. D. Llorach P. Lopez R. C.. (2021). Selective filtering of excitatory inputs to nucleus accumbens by dopamine and serotonin. Proc. Natl. Acad. Sci. U S A 118:e2106648118. 10.1073/pnas.2106648118 34103400 PMC8214692

[B35] Cooper S. E. Kechner M. Caraballo-Pérez D. Kaska S. Robison A. J. Mazei-Robison M. S. (2017). Comparison of chronic physical and emotional social defeat stress effects on mesocorticolimbic circuit activation and voluntary consumption of morphine. Sci. Rep. 7:8445. 10.1038/s41598-017-09106-3 28814751 PMC5559445

[B34] Cooper M. L. Russell M. Skinner J. B. Windle M. (1992). Development and validation of a three-dimensional measure of drinking motives. Psychol. Assess. 4, 123–132. 10.1037/1040-3590.4.2.123

[B36] Cottler L. B. Compton W. M. Mager D. Spitznagel E. L. Janca A. (1992). Posttraumatic stress disorder among substance users from the general population. Am. J. Psychiatry 149, 664–670. 10.1176/ajp.149.5.664 1575258

[B37] Covington H. E. 3rd Miczek K. A. (2005). Intense cocaine self-administration after episodic social defeat stress, but not after aggressive behavior: dissociation from corticosterone activation. Psychopharmacology (Berl) 183, 331–340. 10.1007/s00213-005-0190-5 16249907

[B38] Covington H. E. 3rd Miczek K. A. (2001). Repeated social-defeat stress, cocaine or morphine. Effects on behavioral sensitization and intravenous cocaine self-administration “binges”. Psychopharmacology (Berl) 158, 388–398. 10.1007/s002130100858 11797060

[B39] Covington H. E. Tropea T. F. Rajadhyaksha A. M. Kosofsky B. E. Miczek K. A. (2008). NMDA receptors in the rat VTA: a critical site for social stress to intensify cocaine taking. Psychopharmacology (Berl) 197, 203–216. 10.1007/s00213-007-1024-4 18097654 PMC2664317

[B40] de Wit H. Sayette M. (2018). Considering the context: social factors in responses to drugs in humans. Psychopharmacology (Berl) 235, 935–945. 10.1007/s00213-018-4854-3 29470605 PMC5871591

[B41] Deatherage G. (1972). Effects of housing density on alcohol intake in the rat. Physiol. Behav. 9, 55–57. 10.1016/0031-9384(72)90264-8 4673096

[B42] Der-Avakian A. Mazei-Robison M. S. Kesby J. P. Nestler E. J. Markou A. (2014). Enduring deficits in brain reward function after chronic social defeat in rats: susceptibility, resilience and antidepressant response. Biol. Psychiatry 76, 542–549. 10.1016/j.biopsych.2014.01.013 24576687 PMC4117827

[B43] Di Chiara G. Imperato A. (1988). Drugs abused by humans preferentially increase synaptic dopamine concentrations in the mesolimbic system of freely moving rats. Proc. Natl. Acad. Sci. U S A 85, 5274–5278. 10.1073/pnas.85.14.5274 2899326 PMC281732

[B44] Dölen G. Darvishzadeh A. Huang K. W. Malenka R. C. (2013). Social reward requires coordinated activity of nucleus accumbens oxytocin and serotonin. Nature 501, 179–184. 10.1038/nature12518 24025838 PMC4091761

[B45] Douglas K. R. Chan G. Gelernter J. Arias A. J. Anton R. F. Weiss R. D.. (2010). Adverse childhood events as risk factors for substance dependence: partial mediation by mood and anxiety disorders. Addict. Behav. 35, 7–13. 10.1016/j.addbeh.2009.07.004 19720467 PMC2763992

[B46] Dube S. R. Anda R. F. Felitti V. J. Edwards V. J. Croft J. B. (2002). Adverse childhood experiences and personal alcohol abuse as an adult. Addict. Behav. 27, 713–725. 10.1016/s0306-4603(01)00204-0 12201379

[B47] Fabricius K. Helboe L. Fink-Jensen A. Wörtwein G. Steiniger-Brach B. Sotty F. (2010). Increased dopaminergic activity in socially isolated rats: an electrophysiological study. Neurosci. Lett. 482, 117–122. 10.1016/j.neulet.2010.07.014 20637831

[B48] Ferguson J. N. Aldag J. M. Insel T. R. Young L. J. (2001). Oxytocin in the medial amygdala is essential for social recognition in the mouse. J. Neurosci. 21, 8278–8285. 10.1523/JNEUROSCI.21-20-08278.2001 11588199 PMC6763861

[B49] Folkes O. M. Báldi R. Kondev V. Marcus D. J. Hartley N. D. Turner B. D.. (2020). An endocannabinoid-regulated basolateral amygdala-nucleus accumbens circuit modulates sociability. J. Clin. Invest. 130, 1728–1742. 10.1172/JCI131752 31874107 PMC7108917

[B50] Fonseca M. S. Murakami M. Mainen Z. F. (2015). Activation of dorsal raphe serotonergic neurons promotes waiting but is not reinforcing. Curr. Biol. 25, 306–315. 10.1016/j.cub.2014.12.002 25601545

[B51] Fosnocht A. Q. Lucerne K. E. Ellis A. S. Olimpo N. A. Briand L. A. (2019). Adolescent social isolation increases cocaine seeking in male and female mice. Behav. Brain Res. 359, 589–596. 10.1016/j.bbr.2018.10.007 30296530 PMC6557448

[B52] Fritz M. El Rawas R. Salti A. Klement S. Bardo M. T. Kemmler G.. (2011). Reversal of cocaine-conditioned place preference and mesocorticolimbic Zif268 expression by social interaction in rats. Addict. Biol. 16, 273–284. 10.1111/j.1369-1600.2010.00285.x 21309948

[B54] Funk D. Harding S. Juzytsch W. Lê A. D. (2005). Effects of unconditioned and conditioned social defeat on alcohol self-administration and reinstatement of alcohol seeking in rats. Psychopharmacology (Berl) 183, 341–349. 10.1007/s00213-005-0194-1 16254734

[B53] Funk C. K. Zorrilla E. P. Lee M. J. Rice K. C. Koob G. F. (2007). Corticotropin-releasing factor 1 antagonists selectively reduce ethanol self-administration in ethanol-dependent rats. Biol. Psychiatry 61, 78–86. 10.1016/j.biopsych.2006.03.063 16876134 PMC2741496

[B55] Gage S. H. Sumnall H. R. (2019). Rat Park: How a rat paradise changed the narrative of addiction. Addiction 114, 917–922. 10.1111/add.14481 30367729

[B56] Gilpin N. W. Weiner J. L. (2017). Neurobiology of comorbid post-traumatic stress disorder and alcohol-use disorder. Genes Brain Behav. 16, 15–43. 10.1111/gbb.12349 27749004 PMC5477640

[B57] Gipson C. D. Beckmann J. S. El-Maraghi S. Marusich J. A. Bardo M. T. (2011). Effect of environmental enrichment on escalation of cocaine self-administration in rats. Psychopharmacology (Berl) 214, 557–566. 10.1007/s00213-010-2060-z 21057774 PMC3166517

[B58] Goeldner C. Lutz P. E. Darcq E. Halter T. Clesse D. Ouagazzal A. M.. (2011). Impaired emotional-like behavior and serotonergic function during protracted abstinence from chronic morphine. Biol. Psychiatry 69, 236–244. 10.1016/j.biopsych.2010.08.021 20947067 PMC3014999

[B59] Golden S. A. Covington H. E. Berton O. Russo S. J. (2011). A standardized protocol for repeated social defeat stress in mice. Nat. Protoc. 6, 1183–1191. 10.1038/nprot.2011.361 21799487 PMC3220278

[B60] Green A. R. Mechan A. O. Elliott J. M. O’Shea E. Colado M. I. (2003). The pharmacology and clinical pharmacology of 3,4-methylenedioxymethamphetamine (MDMA, “ecstasy”). Pharmacol. Rev. 55, 463–508. 10.1124/pr.55.3.3 12869661

[B61] Grotewold S. K. Wall V. L. Goodell D. J. Hayter C. Bland S. T. (2014). Effects of cocaine combined with a social cue on conditioned place preference and nucleus accumbens monoamines after isolation rearing in rats. Psychopharmacology (Berl) 231, 3041–3053. 10.1007/s00213-014-3470-0 24553577 PMC4646085

[B62] Gunaydin L. A. Grosenick L. Finkelstein J. C. Kauvar I. V. Fenno L. E. Adhikari A.. (2014). Natural neural projection dynamics underlying social behavior. Cell 157, 1535–1551. 10.1016/j.cell.2014.05.017 24949967 PMC4123133

[B63] Hadaway P. F. Alexander B. K. Coambs R. B. Beyerstein B. (1979). The effect of housing and gender on preference for morphine-sucrose solutions in rats. Psychopharmacology (Berl) 66, 87–91. 10.1007/BF00431995 120547

[B64] Hagino Y. Hall F. S. Uhl G. R. Sora I. Ikeda K. (2021). Dual actions of 5-MeO-DIPT at the serotonin transporter and serotonin 5-HT_1A_ receptor in the mouse striatum and prefrontal cortex. Neuropsychopharmacol. Rep. 41, 91–101. 10.1002/npr2.12161 33547882 PMC8182963

[B65] Hall F. S. Huang S. Fong G. W. Pert A. Linnoila M. (1998). Effects of isolation-rearing on voluntary consumption of ethanol, sucrose and saccharin solutions in Fawn Hooded and Wistar rats. Psychopharmacology (Berl) 139, 210–216. 10.1007/s002130050706 9784075

[B66] Han X. Albrechet-Souza L. Doyle M. R. Shimamoto A. DeBold J. F. Miczek K. A. (2015). Social stress and escalated drug self-administration in mice II. Cocaine and dopamine in the nucleus accumbens. Psychopharmacology (Berl) 232, 1003–1010. 10.1007/s00213-014-3734-8 25216798 PMC4339460

[B67] Han X. DeBold J. F. Miczek K. A. (2017). Prevention and reversal of social stress-escalated cocaine self-administration in mice by intra-VTA CRFR1 antagonism. Psychopharmacology (Berl) 234, 2813–2821. 10.1007/s00213-017-4676-8 28698920 PMC5709170

[B68] Harper K. M. Knapp D. J. Butler R. K. Cook C. A. Criswell H. E. Stuber G. D.. (2019). Amygdala arginine vasopressin modulates chronic ethanol withdrawal anxiety-like behavior in the social interaction task. Alcohol. Clin. Exp. Res. 43, 2134–2143. 10.1111/acer.14163 31386210 PMC6779490

[B69] Heifets B. D. Salgado J. S. Taylor M. D. Hoerbelt P. Cardozo Pinto D. F. Steinberg E. E.. (2019). Distinct neural mechanisms for the prosocial and rewarding properties of MDMA. Sci. Transl. Med. 11:eaaw6435. 10.1126/scitranslmed.aaw6435 31826983 PMC7123941

[B70] Heim C. Nemeroff C. B. (2001). The role of childhood trauma in the neurobiology of mood and anxiety disorders: preclinical and clinical studies. Biol. Psychiatry 49, 1023–1039. 10.1016/s0006-3223(01)01157-x 11430844

[B71] Heyman G. M. (2013). Addiction and choice: theory and new data. Front. Psychiatry 4:31. 10.3389/fpsyt.2013.00031 23653607 PMC3644798

[B72] Hitti F. L. Siegelbaum S. A. (2014). The hippocampal CA2 region is essential for social memory. Nature 508, 88–92. 10.1038/nature13028 24572357 PMC4000264

[B73] Hoffmann J. P. Cerbone F. G. Su S. S. (2000). A growth curve analysis of stress and adolescent drug use. Subst. Use Misuse 35, 687–716. 10.3109/10826080009148417 10807152

[B74] Holly E. N. Boyson C. O. Montagud-Romero S. Stein D. J. Gobrogge K. L. DeBold J. F.. (2016). Episodic social stress-escalated cocaine self-administration: role of phasic and tonic corticotropin releasing factor in the anterior and posterior ventral tegmental area. J. Neurosci. 36, 4093–4105. 10.1523/JNEUROSCI.2232-15.2016 27053215 PMC4821917

[B75] Holly E. N. DeBold J. F. Miczek K. A. (2015). Increased mesocorticolimbic dopamine during acute and repeated social defeat stress: modulation by corticotropin releasing factor receptors in the ventral tegmental area. Psychopharmacology (Berl) 232, 4469–4479. 10.1007/s00213-015-4082-z 26403083 PMC4651830

[B76] Holly E. N. Shimamoto A. Debold J. F. Miczek K. A. (2012). Sex differences in behavioral and neural cross-sensitization and escalated cocaine taking as a result of episodic social defeat stress in rats. Psychopharmacology (Berl) 224, 179–188. 10.1007/s00213-012-2846-2 22926005 PMC3684960

[B77] Hong W. Kim D. W. Anderson D. J. (2014). Antagonistic control of social versus repetitive self-grooming behaviors by separable amygdala neuronal subsets. Cell 158, 1348–1361. 10.1016/j.cell.2014.07.049 25215491 PMC4167378

[B78] Howes S. R. Dalley J. W. Morrison C. H. Robbins T. W. Everitt B. J. (2000). Leftward shift in the acquisition of cocaine self-administration in isolation-reared rats: relationship to extracellular levels of dopamine, serotonin and glutamate in the nucleus accumbens and amygdala-striatal FOS expression. Psychopharmacology (Berl) 151, 55–63. 10.1007/s002130000451 10958117

[B79] Hung L. W. Neuner S. Polepalli J. S. Beier K. T. Wright M. Walsh J. J.. (2017). Gating of social reward by oxytocin in the ventral tegmental area. Science 357, 1406–1411. 10.1126/science.aan4994 28963257 PMC6214365

[B80] Hwa L. S. Holly E. N. DeBold J. F. Miczek K. A. (2016a). Social stress-escalated intermittent alcohol drinking: modulation by CRF-R1 in the ventral tegmental area and accumbal dopamine in mice. Psychopharmacology (Berl) 233, 681–690. 10.1007/s00213-015-4144-2 26576941 PMC4729595

[B81] Hwa L. S. Shimamoto A. Kayyali T. Norman K. J. Valentino R. J. DeBold J. F.. (2016b). Dissociation of μ-opioid receptor and CRF-R1 antagonist effects on escalated ethanol consumption and mPFC serotonin in C57BL/6J mice. Addict. Biol. 21, 111–124. 10.1111/adb.12189 25262980 PMC4377124

[B82] Hyman S. M. Hong K. I. Chaplin T. M. Dabre Z. Comegys A. D. Kimmerling A.. (2009). A stress-coping profile of opioid dependent individuals entering naltrexone treatment: a comparison with healthy controls. Psychol. Addict. Behav. 23, 613–619. 10.1037/a0017324 20025367 PMC2802459

[B83] Ieraci A. Mallei A. Popoli M. (2016). Social isolation stress induces anxious-depressive-like behavior and alterations of neuroplasticity-related genes in adult male mice. Neural Plast. 2016:6212983. 10.1155/2016/6212983 26881124 PMC4736811

[B84] Iñiguez S. D. Flores-Ramirez F. J. Riggs L. M. Alipio J. B. Garcia-Carachure I. Hernandez M. A.. (2018). Vicarious social defeat stress induces depression-related outcomes in female mice. Biol. Psychiatry 83, 9–17. 10.1016/j.biopsych.2017.07.014 28888327 PMC5730407

[B85] Irvine E. E. Bagnalasta M. Marcon C. Motta C. Tessari M. File S. E.. (2001). Nicotine self-administration and withdrawal: modulation of anxiety in the social interaction test in rats. Psychopharmacology (Berl) 153, 315–320. 10.1007/s002130000586 11271403

[B86] Ishikawa J. Ogawa Y. Owada Y. Ishikawa A. (2014). Hyperlocomotor activity and stress vulnerability during adulthood induced by social isolation after early weaning are prevented by voluntary running exercise before normal weaning period. Behav. Brain Res. 264, 197–206. 10.1016/j.bbr.2014.02.007 24534713

[B87] Jones A. C. Schinka K. C. van Dulmen M. H. Bossarte R. M. Swahn M. H. (2011). Changes in loneliness during middle childhood predict risk for adolescent suicidality indirectly through mental health problems. J. Clin. Child Adolesc. Psychol. 40, 818–824. 10.1080/15374416.2011.614585 22023273

[B88] Karkhanis A. N. Alexander N. J. McCool B. A. Weiner J. L. Jones S. R. (2015). Chronic social isolation during adolescence augments catecholamine response to acute ethanol in the basolateral amygdala. Synapse 69, 385–395. 10.1002/syn.21826 25963724 PMC4578716

[B89] Karkhanis A. N. Locke J. L. McCool B. A. Weiner J. L. Jones S. R. (2014). Social isolation rearing increases nucleus accumbens dopamine and norepinephrine responses to acute ethanol in adulthood. Alcohol. Clin. Exp. Res. 38, 2770–2779. 10.1111/acer.12555 25421514 PMC4519347

[B90] Karkhanis A. N. Rose J. H. Weiner J. L. Jones S. R. (2016). Early-life social isolation stress increases kappa opioid receptor responsiveness and downregulates the dopamine system. Neuropsychopharmacology 41, 2263–2274. 10.1038/npp.2016.21 26860203 PMC4946054

[B91] Ko C. Y. Liu Y. P. (2015). Isolation rearing impaired sensorimotor gating but increased pro-inflammatory cytokines and disrupted metabolic parameters in both sexes of rats. Psychoneuroendocrinology 55, 173–183. 10.1016/j.psyneuen.2015.02.007 25770703

[B93] Koob G. F. (2021). Drug addiction: hyperkatifeia/negative reinforcement as a framework for medications development. Pharmacol. Rev. 73, 163–201. 10.1124/pharmrev.120.000083 33318153 PMC7770492

[B92] Koob G. F. Volkow N. D. (2010). Neurocircuitry of addiction. Neuropsychopharmacology 35, 217–238. 10.1038/npp.2009.110 19710631 PMC2805560

[B94] Kosten T. R. Petrakis I. L. (2021). The hidden epidemic of opioid overdoses during the coronavirus disease 2019 pandemic. JAMA Psychiatry 78, 585–586. 10.1001/jamapsychiatry.2020.4148 33377967

[B95] Kudryavtseva N. N. Madorskaya I. A. Bakshtanovskaya I. V. (1991). Social success and voluntary ethanol consumption in mice of C57BL/6J and CBA/Lac strains. Physiol. Behav. 50, 143–146. 10.1016/0031-9384(91)90511-l 1946707

[B96] Lahiri A. K. Bevan M. D. (2020). Dopaminergic transmission rapidly and persistently enhances excitability of d1 receptor-expressing striatal projection neurons. Neuron 106, 277–290.e6. 10.1016/j.neuron.2020.01.028 32075716 PMC7182485

[B97] Laine M. A. Sokolowska E. Dudek M. Callan S. A. Hyytiä P. Hovatta I. (2017). Brain activation induced by chronic psychosocial stress in mice. Sci. Rep. 7:15061. 10.1038/s41598-017-15422-5 29118417 PMC5678090

[B98] Lalanne L. Ayranci G. Filliol D. Gavériaux-Ruff C. Befort K. Kieffer B. L.. (2017). Kappa opioid receptor antagonism and chronic antidepressant treatment have beneficial activities on social interactions and grooming deficits during heroin abstinence. Addict. Biol. 22, 1010–1021. 10.1111/adb.12392 27001273 PMC5590636

[B99] Land B. B. Bruchas M. R. Schattauer S. Giardino W. J. Aita M. Messinger D.. (2009). Activation of the kappa opioid receptor in the dorsal raphe nucleus mediates the aversive effects of stress and reinstates drug seeking. Proc. Natl. Acad. Sci. U S A 106, 19168–19173. 10.1073/pnas.0910705106 19864633 PMC2776420

[B100] Lawson K. M. Back S. E. Hartwell K. J. Moran-Santa Maria M. Brady K. T. (2013). A comparison of trauma profiles among individuals with prescription opioid, nicotine, or cocaine dependence. Am. J. Addict. 22, 127–131. 10.1111/j.1521-0391.2013.00319.x 23414497 PMC3681508

[B101] Leclercq S. Le Roy T. Furgiuele S. Coste V. Bindels L. B. Leyrolle Q.. (2020). Gut microbiota-induced changes in β-hydroxybutyrate metabolism are linked to altered sociability and depression in alcohol use disorder. Cell Rep. 33:108238. 10.1016/j.celrep.2020.108238 33053357

[B102] Lee J. S. Lee S. B. Kim D. W. Shin N. Jeong S. J. Yang C. H.. (2021). Social isolation-related depression accelerates ethanol intake *via* microglia-derived neuroinflammation. Sci. Adv. 7:eabj3400. 10.1126/sciadv.abj3400 34739315 PMC8570606

[B103] Lemos J. C. Wanat M. J. Smith J. S. Reyes B. A. Hollon N. G. Van Bockstaele E. J.. (2012). Severe stress switches CRF action in the nucleus accumbens from appetitive to aversive. Nature 490, 402–406. 10.1038/nature11436 22992525 PMC3475726

[B104] Li Y. Simmler L. D. Van Zessen R. Flakowski J. Wan J. X. Deng F.. (2021). Synaptic mechanism underlying serotonin modulation of transition to cocaine addiction. Science 373, 1252–1256. 10.1126/science.abi9086 34516792 PMC8817894

[B105] Litvin Y. Murakami G. Pfaff D. W. (2011). Effects of chronic social defeat on behavioral and neural correlates of sociality: vasopressin, oxytocin and the vasopressinergic V1b receptor. Physiol. Behav. 103, 393–403. 10.1016/j.physbeh.2011.03.007 21397619

[B107] Liu Y. P. Kao Y. C. Tung C. S. (2011). Critical period exists in the effects of isolation rearing on sensorimotor gating function but not locomotor activity in rat. Prog. Neuropsychopharmacol. Biol. Psychiatry 35, 1068–1073. 10.1016/j.pnpbp.2011.03.002 21396422

[B108] Liu Z. Lin R. Luo M. (2020). Reward contributions to serotonergic functions. Annu. Rev. Neurosci. 43, 141–162. 10.1146/annurev-neuro-093019-112252 32640931

[B106] Liu Y. Wang Z. X. (2003). Nucleus accumbens oxytocin and dopamine interact to regulate pair bond formation in female prairie voles. Neuroscience 121, 537–544. 10.1016/s0306-4522(03)00555-4 14568015

[B109] Lodge D. J. Grace A. A. (2005). Acute and chronic corticotropin-releasing factor 1 receptor blockade inhibits cocaine-induced dopamine release: correlation with dopamine neuron activity. J. Pharmacol. Exp. Ther. 314, 201–206. 10.1124/jpet.105.084913 15784652

[B110] Lowery-Gionta E. G. Marcinkiewcz C. A. Kash T. L. (2015). Functional alterations in the dorsal raphe nucleus following acute and chronic ethanol exposure. Neuropsychopharmacology 40, 590–600. 10.1038/npp.2014.205 25120075 PMC4289946

[B111] Lu L. Liu Z. Huang M. Zhang Z. (2003). Dopamine-dependent responses to cocaine depend on corticotropin-releasing factor receptor subtypes. J. Neurochem. 84, 1378–1386. 10.1046/j.1471-4159.2003.01635.x 12614338

[B112] Lutz P. E. Pradhan A. A. Goeldner C. Kieffer B. L. (2011). Sequential and opposing alterations of 5-HT(1A) receptor function during withdrawal from chronic morphine. Eur. Neuropsychopharmacol. 21, 835–840. 10.1016/j.euroneuro.2011.02.002 21402471 PMC3149735

[B113] Maier S. F. Watkins L. R. (2005). Stressor controllability and learned helplessness: the roles of the dorsal raphe nucleus, serotonin and corticotropin-releasing factor. Neurosci. Biobehav. Rev. 29, 829–841. 10.1016/j.neubiorev.2005.03.021 15893820

[B114] Manvich D. F. Stowe T. A. Godfrey J. R. Weinshenker D. (2016). A method for psychosocial stress-induced reinstatement of cocaine seeking in rats. Biol. Psychiatry 79, 940–946. 10.1016/j.biopsych.2015.07.002 26257242 PMC4706515

[B115] Marcinkiewcz C. A. Dorrier C. E. Lopez A. J. Kash T. L. (2015). Ethanol induced adaptations in 5-HT2c receptor signaling in the bed nucleus of the stria terminalis: implications for anxiety during ethanol withdrawal. Neuropharmacology 89, 157–167. 10.1016/j.neuropharm.2014.09.003 25229718 PMC4469779

[B116] Marks-Kaufman R. Lewis M. J. (1984). Early housing experience modifies morphine self-administration and physical dependence in adult rats. Addict. Behav. 9, 235–243. 10.1016/0306-4603(84)90015-7 6541860

[B117] McCool B. A. Chappell A. M. (2009). Early social isolation in male long-evans rats alters both appetitive and consummatory behaviors expressed during operant ethanol self-administration. Alcohol. Clin. Exp. Res. 33, 273–282. 10.1111/j.1530-0277.2008.00830.x 19032581 PMC2633417

[B118] McDevitt R. A. Marino R. A. M. Tejeda H. A. Bonci A. (2021). Serotonergic inhibition of responding for conditioned but not primary reinforcers. Pharmacol. Biochem. Behav. 205:173186. 10.1016/j.pbb.2021.173186 33836219 PMC9635562

[B119] McGregor I. S. Bowen M. T. (2012). Breaking the loop: oxytocin as a potential treatment for drug addiction. Horm. Behav. 61, 331–339. 10.1016/j.yhbeh.2011.12.001 22198308

[B120] McKenzie-Quirk S. D. Miczek K. A. (2008). Social rank and social separation as determinants of alcohol drinking in squirrel monkeys. Psychopharmacology (Berl) 201, 137–145. 10.1007/s00213-008-1256-y 18641974 PMC4371730

[B121] McLaughlin J. P. Li S. Valdez J. Chavkin T. A. Chavkin C. (2006). Social defeat stress-induced behavioral responses are mediated by the endogenous kappa opioid system. Neuropsychopharmacology 31, 1241–1248. 10.1038/sj.npp.1300872 16123746 PMC2096774

[B122] Meyers R. J. Roozen H. G. Smith J. E. (2011). The community reinforcement approach: an update of the evidence. Alcohol. Res. Health 33, 380–388. 10.1007/978-90-313-9756-3 23580022 PMC3860533

[B123] Miczek K. A. Mutschler N. H. van Erp A. M. Blank A. D. McInerney S. C. (1999a). d-amphetamine “cue” generalizes to social defeat stress: behavioral sensitization and attenuated accumbens dopamine. Psychopharmacology (Berl) 147, 190–199. 10.1007/s002130051160 10591887

[B124] Miczek K. A. Nikulina E. Kream R. M. Carter G. Espejo E. F. (1999b). Behavioral sensitization to cocaine after a brief social defeat stress: c-fos expression in the PAG. Psychopharmacology (Berl) 141, 225–234. 10.1007/s002130050829 10027503

[B125] Miczek K. A. Nikulina E. M. Shimamoto A. Covington H. E. (2011). Escalated or suppressed cocaine reward, tegmental BDNF and accumbal dopamine caused by episodic versus continuous social stress in rats. J. Neurosci. 31, 9848–9857. 10.1523/JNEUROSCI.0637-11.2011 21734276 PMC3144494

[B126] Miczek K. A. Yap J. J. Covington H. E. (2008). Social stress, therapeutics and drug abuse: preclinical models of escalated and depressed intake. Pharmacol. Ther. 120, 102–128. 10.1016/j.pharmthera.2008.07.006 18789966 PMC2713609

[B127] Miyazaki K. W. Miyazaki K. Tanaka K. F. Yamanaka A. Takahashi A. Tabuchi S.. (2014). Optogenetic activation of dorsal raphe serotonin neurons enhances patience for future rewards. Curr. Biol. 24, 2033–2040. 10.1016/j.cub.2014.07.041 25155504

[B128] Montagud-Romero S. Aguilar M. A. Maldonado C. Manzanedo C. Miñarro J. Rodríguez-Arias M. (2015). Acute social defeat stress increases the conditioned rewarding effects of cocaine in adult but not in adolescent mice. Pharmacol. Biochem. Behav. 135, 1–12. 10.1016/j.pbb.2015.05.008 25989047

[B129] Montagud-Romero S. Reguilon M. D. Roger-Sanchez C. Pascual M. Aguilar M. A. Guerri C.. (2016). Role of dopamine neurotransmission in the long-term effects of repeated social defeat on the conditioned rewarding effects of cocaine. Prog. Neuropsychopharmacol. Biol. Psychiatry 71, 144–154. 10.1016/j.pnpbp.2016.07.008 27476156

[B130] Morisot N. Monier R. Le Moine C. Millan M. J. Contarino A. (2018). Corticotropin-releasing factor receptor 2-deficiency eliminates social behaviour deficits and vulnerability induced by cocaine. Br. J. Pharmacol. 175, 1504–1518. 10.1111/bph.14159 29406581 PMC5900993

[B131] Morley K. C. Gallate J. E. Hunt G. E. Mallet P. E. McGregor I. S. (2001). Increased anxiety and impaired memory in rats 3 months after administration of 3,4-methylenedioxymethamphetamine (“ecstasy”). Eur. J. Pharmacol. 433, 91–99. 10.1016/s0014-2999(01)01512-6 11755138

[B132] Murugan M. Jang H. J. Park M. Miller E. M. Cox J. Taliaferro J. P.. (2017). Combined social and spatial coding in a descending projection from the prefrontal cortex. Cell 171, 1663–1677.e16. 10.1016/j.cell.2017.11.002 29224779 PMC5889923

[B133] Nardou R. Lewis E. M. Rothhaas R. Xu R. Yang A. Boyden E.. (2019). Oxytocin-dependent reopening of a social reward learning critical period with MDMA. Nature 569, 116–120. 10.1038/s41586-019-1075-9 30944474

[B134] Nelson B. S. Sequeira M. K. Schank J. R. (2018). Bidirectional relationship between alcohol intake and sensitivity to social defeat: association with Tacr1 and Avp expression. Addict. Biol. 23, 142–153. 10.1111/adb.12494 28150369 PMC5538906

[B135] Newman E. L. Albrechet-Souza L. Andrew P. M. Auld J. G. Burk K. C. Hwa L. S.. (2018a). Persistent escalation of alcohol consumption by mice exposed to brief episodes of social defeat stress: suppression by CRF-R1 antagonism. Psychopharmacology (Berl) 235, 1807–1820. 10.1007/s00213-018-4905-9 29696309 PMC6168197

[B139] Newman E. L. Leonard M. Z. Arena D. T. de Almeida R. M. M. Miczek K. A. (2018b). Social defeat stress and escalation of cocaine and alcohol consumption: focus on CRF. Neurobiol. Stress 9, 151–.165. 10.1016/j.ynstr.2018.09.007 30450381 PMC6236516

[B136] Newman E. L. Chu A. Bahamón B. Takahashi A. Debold J. F. Miczek K. A. (2012). NMDA receptor antagonism: escalation of aggressive behavior in alcohol-drinking mice. Psychopharmacology (Berl) 224, 167–177. 10.1007/s00213-012-2734-9 22588250 PMC3694321

[B137] Newman E. L. Covington H. E. Leonard M. Z. Burk K. Miczek K. A. (2021). Hypoactive thalamic Crh+ cells in a female mouse model of alcohol drinking after social trauma. Biol. Psychiatry 90, 563–574. 10.1016/j.biopsych.2021.05.022 34281710 PMC8463500

[B138] Newman E. L. Covington H. E. Suh J. Bicakci M. B. Ressler K. J. DeBold J. F.. (2019). Fighting females: neural and behavioral onsequences of social defeat stress in female mice. Biol. Psychiatry 86, 657–668. 10.1016/j.biopsych.2019.05.005 31255250 PMC6788975

[B140] Niedhammer I. David S. Degioanni S. Drummond A. Philip P. Acquarone D.. (2011). Workplace bullying and psychotropic drug use: the mediating role of physical and mental health status. Ann. Occup. Hyg. 55, 152–163. 10.1093/annhyg/meq086 21177264 PMC3313910

[B141] Nikulina E. M. Hammer R. P. Miczek K. A. Kream R. M. (1999). Social defeat stress increases expression of mu-opioid receptor mRNA in rat ventral tegmental area. Neuroreport 10, 3015–3019. 10.1097/00001756-199909290-00026 10549815

[B142] Nikulina E. M. Miczek K. A. Hammer R. P. (2005). Prolonged effects of repeated social defeat stress on mRNA expression and function of mu-opioid receptors in the ventral tegmental area of rats. Neuropsychopharmacology 30, 1096–1103. 10.1038/sj.npp.1300658 15668724

[B143] Nocjar C. Zhang J. Feng P. Panksepp J. (2012). The social defeat animal model of depression shows diminished levels of orexin in mesocortical regions of the dopamine system and of dynorphin and orexin in the hypothalamus. Neuroscience 218, 138–153. 10.1016/j.neuroscience.2012.05.033 22626650

[B144] Norman K. J. Seiden J. A. Klickstein J. A. Han X. Hwa L. S. DeBold J. F.. (2015). Social stress and escalated drug self-administration in mice I. Alcohol and corticosterone. Psychopharmacology (Berl) 232, 991–1001. 10.1007/s00213-014-3733-9 25242256 PMC4339510

[B145] Oakly A. C. Brox B. W. Schenk S. Ellenbroek B. A. (2014). A genetic deletion of the serotonin transporter greatly enhances the reinforcing properties of MDMA in rats. Mol. Psychiatry 19, 534–535. 10.1038/mp.2013.75 23711978

[B146] Overstreet D. H. Knapp D. J. Moy S. S. Breese G. R. (2003). A 5-HT1A agonist and a 5-HT2c antagonist reduce social interaction deficit induced by multiple ethanol withdrawals in rats. Psychopharmacology (Berl) 167, 344–352. 10.1007/s00213-003-1425-y 12677355 PMC2865243

[B147] Pearson M. Sweeting H. West P. Young R. Gordon J. Turner K. (2006). Adolescent substance use in different social and peer contexts: a social network analysis. Drugs 13, 519–536. 10.1080/09687630600828912

[B148] Pickens C. L. Airavaara M. Theberge F. Fanous S. Hope B. T. Shaham Y. (2011). Neurobiology of the incubation of drug craving. Trends Neurosci. 34, 411–420. 10.1016/j.tins.2011.06.001 21764143 PMC3152666

[B149] Pirino B. E. Spodnick M. B. Gargiulo A. T. Curtis G. R. Barson J. R. Karkhanis A. N. (2020). Kappa-opioid receptor-dependent changes in dopamine and anxiety-like or approach-avoidance behavior occur differentially across the nucleus accumbens shell rostro-caudal axis. Neuropharmacology 181:108341. 10.1016/j.neuropharm.2020.108341 33011200 PMC8424943

[B150] Piskorowski R. A. Nasrallah K. Diamantopoulou A. Mukai J. Hassan S. I. Siegelbaum S. A.. (2016). Age-dependent specific changes in area CA2 of the hippocampus and social memory deficit in a mouse model of the 22q11.2 deletion syndrome. Neuron 89, 163–176. 10.1016/j.neuron.2015.11.036 26748091 PMC4706988

[B151] Quadros I. M. Hwa L. S. Shimamoto A. Carlson J. DeBold J. F. Miczek K. A. (2014). Prevention of alcohol-heightened aggression by CRF-R1 antagonists in mice: critical role for DRN-PFC serotonin pathway. Neuropsychopharmacology 39, 2874–2883. 10.1038/npp.2014.139 24917195 PMC4200498

[B152] Rau A. R. Chappell A. M. Butler T. R. Ariwodola O. J. Weiner J. L. (2015). Increased basolateral amygdala pyramidal cell excitability may contribute to the anxiogenic phenotype induced by chronic early-life stress. J. Neurosci. 35, 9730–9740. 10.1523/JNEUROSCI.0384-15.2015 26134655 PMC4571506

[B153] Resendez S. L. Keyes P. C. Day J. J. Hambro C. Austin C. J. Maina F. K.. (2016). Dopamine and opioid systems interact within the nucleus accumbens to maintain monogamous pair bonds. eLife 5:e15325. 10.7554/eLife.15325 27371827 PMC4972541

[B154] Ribeiro Do Couto B. Aguilar M. A. Manzanedo C. Rodríguez-Arias M. Armario A. Miñarro J. (2006). Social stress is as effective as physical stress in reinstating morphine-induced place preference in mice. Psychopharmacology (Berl) 185, 459–470. 10.1007/s00213-006-0345-z 16555060

[B155] Riga D. Schmitz L. J. van der Harst J. E. van Mourik Y. Hoogendijk W. J. Smit A. B.. (2014). A sustained depressive state promotes a guanfacine reversible susceptibility to alcohol seeking in rats. Neuropsychopharmacology 39, 1115–1124. 10.1038/npp.2013.311 24192553 PMC3957105

[B156] Rincón-Cortés M. Gagnon K. G. Dollish H. K. Grace A. A. (2018). Diazepam reverses increased anxiety-like behavior, social behavior deficit and dopamine dysregulation following withdrawal from acute amphetamine. Neuropsychopharmacology 43, 2418–2425. 10.1038/s41386-018-0123-8 29959439 PMC6180061

[B157] Rivera-Irizarry J. K. Skelly M. J. Pleil K. E. (2020). Social isolation stress in adolescence, but not adulthood, produces hypersocial behavior in adult male and female C57BL/6J Mice. Front. Behav. Neurosci. 14:129. 10.3389/fnbeh.2020.00129 32792924 PMC7394086

[B160] Robinson T. E. Berridge K. C. (1993). The neural basis of drug craving: an incentive-sensitization theory of addiction. Brain Res. Brain Res. Rev. 18, 247–291. 10.1016/0165-0173(93)90013-p 8401595

[B158] Robinson D. L. Heien M. L. Wightman R. M. (2002). Frequency of dopamine concentration transients increases in dorsal and ventral striatum of male rats during introduction of conspecifics. J. Neurosci. 22, 10477–10486. 10.1523/JNEUROSCI.22-23-10477.2002 12451147 PMC6758730

[B159] Robinson D. L. Zitzman D. L. Smith K. J. Spear L. P. (2011). Fast dopamine release events in the nucleus accumbens of early adolescent rats. Neuroscience 176, 296–307. 10.1016/j.neuroscience.2010.12.016 21182904 PMC3061289

[B161] Rusby J. C. Forrester K. K. Biglan A. Metzler C. W. (2005). Relationships between peer harassment and adolescent problem behaviors. J. Early Adoles. 25, 453–477. 10.1177/0272431605279837

[B162] Ryabinin A. E. Bachtell R. K. Heinrichs S. C. Lee S. Rivier C. Olive M. F.. (2002). The corticotropin-releasing factor/urocortin system and alcohol. Alcohol. Clin. Exp. Res. 26, 714–722. 10.1111/j.1530-0277.2002.tb02596.x 12045481

[B163] Sapolsky R. M. (2015). Stress and the brain: individual variability and the inverted-U. Nat. Neurosci. 18, 1344–1346. 10.1038/nn.4109 26404708

[B164] Scheier L. M. Botvin G. J. Diaz T. Griffin K. W. (1999). Social skills, competence and drug refusal efficacy as predictors of adolescent alcohol use. J. Drug Educ. 29, 251–278. 10.2190/M3CT-WWJM-5JAQ-WP15 10645126

[B165] Schenk S. Gorman K. Amit Z. (1990). Age-dependent effects of isolation housing on the self-administration of ethanol in laboratory rats. Alcohol 7, 321–326. 10.1016/0741-8329(90)90090-y 2390208

[B166] Schenk S. Lacelle G. Gorman K. Amit Z. (1987). Cocaine self-administration in rats influenced by environmental conditions: implications for the etiology of drug abuse. Neurosci. Lett. 81, 227–231. 10.1016/0304-3940(87)91003-2 3696469

[B167] Schindler A. G. Messinger D. I. Smith J. S. Shankar H. Gustin R. M. Schattauer S. S.. (2012). Stress produces aversion and potentiates cocaine reward by releasing endogenous dynorphins in the ventral striatum to locally stimulate serotonin reuptake. J. Neurosci. 32, 17582–17596. 10.1523/JNEUROSCI.3220-12.2012 23223282 PMC3523715

[B168] Shahrokh D. K. Zhang T. Y. Diorio J. Gratton A. Meaney M. J. (2010). Oxytocin-dopamine interactions mediate variations in maternal behavior in the rat. Endocrinology 151, 2276–2286. 10.1210/en.2009-1271 20228171 PMC2869254

[B169] Shamay-Tsoory S. G. Abu-Akel A. (2016). The social salience hypothesis of oxytocin. Biol. Psychiatry 79, 194–202. 10.1016/j.biopsych.2015.07.020 26321019

[B170] Shimamoto A. Holly E. N. Boyson C. O. DeBold J. F. Miczek K. A. (2015). Individual differences in anhedonic and accumbal dopamine responses to chronic social stress and their link to cocaine self-administration in female rats. Psychopharmacology (Berl) 232, 825–834. 10.1007/s00213-014-3725-9 25178816 PMC4310791

[B171] Simpson J. Kelly J. P. (2012). The effects of isolated and enriched housing conditions on baseline and drug-induced behavioural responses in the male rat. Behav. Brain Res. 234, 175–183. 10.1016/j.bbr.2012.06.015 22732260

[B172] Sinha R. (2001). How does stress increase risk of drug abuse and relapse. Psychopharmacology (Berl) 158, 343–359. 10.1007/s002130100917 11797055

[B173] Skelly M. J. Chappell A. E. Carter E. Weiner J. L. (2015). Adolescent social isolation increases anxiety-like behavior and ethanol intake and impairs fear extinction in adulthood: possible role of disrupted noradrenergic signaling. Neuropharmacology 97, 149–159. 10.1016/j.neuropharm.2015.05.025 26044636 PMC4537360

[B175] Smith M. A. (2012). Peer influences on drug self-administration: social facilitation and social inhibition of cocaine intake in male rats. Psychopharmacology (Berl) 224, 81–90. 10.1007/s00213-012-2737-6 22588251 PMC3752977

[B174] Smith M. A. Lacy R. T. Strickland J. C. (2014). The effects of social learning on the acquisition of cocaine self-administration. Drug Alcohol. Depend. 141, 1–8. 10.1016/j.drugalcdep.2014.04.025 24878249 PMC4102004

[B176] Song Z. Borland J. M. Larkin T. E. O’Malley M. Albers H. E. (2016). Activation of oxytocin receptors, but not arginine-vasopressin V1a receptors, in the ventral tegmental area of male Syrian hamsters is essential for the reward-like properties of social interactions. Psychoneuroendocrinology 74, 164–172. 10.1016/j.psyneuen.2016.09.001 27632574 PMC6417503

[B177] Spanagel R. Herz A. Shippenberg T. S. (1992). Opposing tonically active endogenous opioid systems modulate the mesolimbic dopaminergic pathway. Proc. Natl. Acad. Sci. U S A 89, 2046–2050. 10.1073/pnas.89.6.2046 1347943 PMC48593

[B178] Stelly C. E. Pomrenze M. B. Cook J. B. Morikawa H. (2016). Repeated social defeat stress enhances glutamatergic synaptic plasticity in the VTA and cocaine place conditioning. eLife 5:e15448. 10.7554/eLife.15448 27374604 PMC4931908

[B179] Strang J. Volkow N. D. Degenhardt L. Hickman M. Johnson K. Koob G. F.. (2020). Opioid use disorder. Nat. Rev. Dis. Primers 6:3. 10.1038/s41572-019-0137-5 31919349

[B180] Tao R. Ba H. Chen J. Liu M. Pan H. Li X.. (2020). Phylodynamic analysis of two amino acid substitutions in the hemagglutinin protein of canine distemper virus strains detected in fur-bearing animals in China. Virus Genes 56, 58–66. 10.1007/s11262-019-01720-9 31802380

[B181] Tharp-Taylor S. Haviland A. D’Amico E. J. (2009). Victimization from mental and physical bullying and substance use in early adolescence. Addict. Behav. 34, 561–567. 10.1016/j.addbeh.2009.03.012 19398162 PMC2707251

[B182] Tidey J. W. Miczek K. A. (1997). Acquisition of cocaine self-administration after social stress: role of accumbens dopamine. Psychopharmacology (Berl) 130, 203–212. 10.1007/s002130050230 9151353

[B183] Tidey J. W. Miczek K. A. (1996). Social defeat stress selectively alters mesocorticolimbic dopamine release: an *in vivo* microdialysis study. Brain Res. 721, 140–149. 10.1016/0006-8993(96)00159-x 8793094

[B184] Tops M. Koole S. L. IJzerman H. Buisman-Pijlman F. T. (2014). Why social attachment and oxytocin protect against addiction and stress: insights from the dynamics between ventral and dorsal corticostriatal systems. Pharmacol. Biochem. Behav. 119, 39–48. 10.1016/j.pbb.2013.07.015 23916423

[B185] Tovar-Díaz J. Pomrenze M. B. Kan R. Pahlavan B. Morikawa H. (2018). Cooperative CRF and α1 adrenergic signaling in the VTA promotes NMDA plasticity and drives social stress enhancement of cocaine conditioning. Cell Rep. 22, 2756–2766. 10.1016/j.celrep.2018.02.039 29514102 PMC5877815

[B186] Ungless M. A. Singh V. Crowder T. L. Yaka R. Ron D. Bonci A. (2003). Corticotropin-releasing factor requires CRF binding protein to potentiate NMDA receptors *via* CRF receptor 2 in dopamine neurons. Neuron 39, 401–407. 10.1016/s0896-6273(03)00461-6 12895416

[B187] Valdez G. R. Roberts A. J. Chan K. Davis H. Brennan M. Zorrilla E. P.. (2002). Increased ethanol self-administration and anxiety-like behavior during acute ethanol withdrawal and protracted abstinence: regulation by corticotropin-releasing factor. Alcohol. Clin. Exp. Res. 26, 1494–1501. 10.1097/01.ALC.0000033120.51856.F0 12394282

[B188] Valentino R. J. Van Bockstaele E. (2008). Convergent regulation of locus coeruleus activity as an adaptive response to stress. Eur. J. Pharmacol. 583, 194–203. 10.1016/j.ejphar.2007.11.062 18255055 PMC2349983

[B189] Valentinova K. Tchenio A. Trusel M. Clerke J. A. Lalive A. L. Tzanoulinou S.. (2019). Morphine withdrawal recruits lateral habenula cytokine signaling to reduce synaptic excitation and sociability. Nat. Neurosci. 22, 1053–1056. 10.1038/s41593-019-0421-4 31209376

[B190] Vanderschuren L. J. Kalivas P. W. (2000). Alterations in dopaminergic and glutamatergic transmission in the induction and expression of behavioral sensitization: a critical review of preclinical studies. Psychopharmacology (Berl) 151, 99–120. 10.1007/s002130000493 10972458

[B191] Venniro M. Banks M. L. Heilig M. Epstein D. H. Shaham Y. (2020a). Improving translation of animal models of addiction and relapse by reverse translation. Nat. Rev. Neurosci. 21, 625–643. 10.1038/s41583-020-0378-z 33024318

[B193] Venniro M. Russell T. I. Ramsey L. A. Richie C. T. Lesscher H. M. B. Giovanetti S. M.. (2020b). Abstinence-dependent dissociable central amygdala microcircuits control drug craving. Proc. Natl. Acad. Sci. U S A 117, 8126–8134. 10.1073/pnas.2001615117 32205443 PMC7148559

[B192] Venniro M. Panlilio L. V. Epstein D. H. Shaham Y. (2021). The protective effect of operant social reward on cocaine self-administration, choice and relapse is dependent on delay and effort for the social reward. Neuropsychopharmacology 46, 2350–2357. 10.1038/s41386-021-01148-6 34400784 PMC8580997

[B194] Venniro M. Russell T. I. Zhang M. Shaham Y. (2019). Operant social reward decreases incubation of heroin craving in male and female rats. Biol. Psychiatry 86, 848–856. 10.1016/j.biopsych.2019.05.018 31326085 PMC8383184

[B195] Venniro M. Zhang M. Caprioli D. Hoots J. K. Golden S. A. Heins C.. (2018). Volitional social interaction prevents drug addiction in rat models. Nat. Neurosci. 21, 1520–1529. 10.1038/s41593-018-0246-6 30323276 PMC7386559

[B196] Volkow N. D. Baler R. D. Goldstein R. Z. (2011). Addiction: pulling at the neural threads of social behaviors. Neuron 69, 599–602. 10.1016/j.neuron.2011.01.027 21338873 PMC3188411

[B197] Wallace B. C. (1989). Psychological and environmental determinants of relapse in crack cocaine smokers. J. Subst. Abuse Treat. 6, 95–106. 10.1016/0740-5472(89)90036-6 2746717

[B198] Wallace D. L. Han M. H. Graham D. L. Green T. A. Vialou V. Iñiguez S. D.. (2009). CREB regulation of nucleus accumbens excitability mediates social isolation-induced behavioral deficits. Nat. Neurosci. 12, 200–209. 10.1038/nn.2257 19151710 PMC2721778

[B199] Walsh J. J. Christoffel D. J. Heifets B. D. Ben-Dor G. A. Selimbeyoglu A. Hung L. W.. (2018). 5-HT release in nucleus accumbens rescues social deficits in mouse autism model. Nature 560, 589–594. 10.1038/s41586-018-0416-4 30089910 PMC8164568

[B200] Wanat M. J. Bonci A. Phillips P. E. (2013). CRF acts in the midbrain to attenuate accumbens dopamine release to rewards but not their predictors. Nat. Neurosci. 16, 383–385. 10.1038/nn.3335 23416448 PMC3609940

[B201] Wanat M. J. Hopf F. W. Stuber G. D. Phillips P. E. Bonci A. (2008). Corticotropin-releasing factor increases mouse ventral tegmental area dopamine neuron firing through a protein kinase C-dependent enhancement of Ih. J. Physiol. 586, 2157–2170. 10.1113/jphysiol.2007.150078 18308824 PMC2465205

[B202] Welsch L. Bailly J. Darcq E. Kieffer B. L. (2020). The negative affect of protracted opioid abstinence: progress and perspectives from rodent models. Biol. Psychiatry 87, 54–63. 10.1016/j.biopsych.2019.07.027 31521334 PMC6898775

[B203] Whitaker L. R. Degoulet M. Morikawa H. (2013). Social deprivation enhances VTA synaptic plasticity and drug-induced contextual learning. Neuron 77, 335–345. 10.1016/j.neuron.2012.11.022 23352169 PMC3559005

[B204] Wills T. A. Knapp D. J. Overstreet D. H. Breese G. R. (2009). Sensitization, duration and pharmacological blockade of anxiety-like behavior following repeated ethanol withdrawal in adolescent and adult rats. Alcohol. Clin. Exp. Res. 33, 455–463. 10.1111/j.1530-0277.2008.00856.x 19120055 PMC2847263

[B205] Wolffgramm J. (1990). Free choice ethanol intake of laboratory rats under different social conditions. Psychopharmacology (Berl) 101, 233–239. 10.1007/BF02244132 2349365

[B206] Wongtongkam N. Ward P. R. Day A. Winefield A. H. (2015). Exploring family and community involvement to protect Thai youths from alcohol and illegal drug abuse. J. Addict. Dis. 34, 112–121. 10.1080/10550887.2014.975616 25491376

[B207] Wongwitdecha N. Marsden C. A. (1996). Effect of social isolation on the reinforcing properties of morphine in the conditioned place preference test. Pharmacol. Biochem. Behav. 53, 531–534. 10.1016/0091-3057(95)02046-2 8866951

[B209] Wu Y. E. Dang J. Kingsbury L. Zhang M. Sun F. Hu R. K.. (2021). Neural control of affiliative touch in prosocial interaction. Nature 599, 262–267. 10.1038/s41586-021-03962-w 34646019 PMC8605624

[B208] Wu X. Pang G. Zhang Y. M. Li G. Xu S. Dong L.. (2015). Activation of serotonin 5-HT(2C) receptor suppresses behavioral sensitization and naloxone-precipitated withdrawal symptoms in heroin-treated mice. Neurosci. Lett. 607, 23–28. 10.1016/j.neulet.2015.09.013 26375926 PMC4631619

[B210] Xiao L. Priest M. F. Nasenbeny J. Lu T. Kozorovitskiy Y. (2017). Biased oxytocinergic modulation of midbrain dopamine systems. Neuron 95, 368–384.e5. 10.1016/j.neuron.2017.06.003 28669546 PMC7881764

[B211] Xu S. Das G. Hueske E. Tonegawa S. (2017). Dorsal raphe serotonergic neurons control intertemporal choice under trade-off. Curr. Biol. 27, 3111–3119.e3. 10.1016/j.cub.2017.09.008 28988863 PMC5691357

[B212] Yavich L. Tiihonen J. (2000). Ethanol modulates evoked dopamine release in mouse nucleus accumbens: dependence on social stress and dose. Eur. J. Pharmacol. 401, 365–373. 10.1016/s0014-2999(00)00456-8 10936495

[B213] Yizhar O. Fenno L. E. Prigge M. Schneider F. Davidson T. J. O’Shea D. J.. (2011). Neocortical excitation/inhibition balance in information processing and social dysfunction. Nature 477, 171–178. 10.1038/nature10360 21796121 PMC4155501

[B214] Yorgason J. T. Calipari E. S. Ferris M. J. Karkhanis A. N. Fordahl S. C. Weiner J. L.. (2016). Social isolation rearing increases dopamine uptake and psychostimulant potency in the striatum. Neuropharmacology 101, 471–479. 10.1016/j.neuropharm.2015.10.025 26525189 PMC4681685

[B215] Yorgason J. T. España R. A. Konstantopoulos J. K. Weiner J. L. Jones S. R. (2013). Enduring increases in anxiety-like behavior and rapid nucleus accumbens dopamine signaling in socially isolated rats. Eur. J. Neurosci. 37, 1022–1031. 10.1111/ejn.12113 23294165 PMC3746484

[B216] Young L. J. Wang Z. (2004). The neurobiology of pair bonding. Nat. Neurosci. 7, 1048–1054. 10.1038/nn1327 15452576

[B217] Zakharova E. Miller J. Unterwald E. Wade D. Izenwasser S. (2009). Social and physical environment alter cocaine conditioned place preference and dopaminergic markers in adolescent male rats. Neuroscience 163, 890–897. 10.1016/j.neuroscience.2009.06.068 19580849 PMC2746859

[B218] Zanos P. Georgiou P. Gonzalez L. R. Hourani S. Chen Y. Kitchen I.. (2016). Emotional impairment and persistent upregulation of mGlu5 receptor following morphine abstinence: implications of an mGlu5-MOPr interaction. Int. J. Neuropsychopharmacol. 19:pyw011. 10.1093/ijnp/pyw011 26861145 PMC4966274

[B219] Zanos P. Georgiou P. Wright S. R. Hourani S. M. Kitchen I. Winsky-Sommerer R.. (2014). The oxytocin analogue carbetocin prevents emotional impairment and stress-induced reinstatement of opioid-seeking in morphine-abstinent mice. Neuropsychopharmacology 39, 855–865. 10.1038/npp.2013.285 24129263 PMC3924520

[B220] Zelikowsky M. Ding K. Anderson D. J. (2018a). Neuropeptidergic control of an internal brain state produced by prolonged social isolation stress. Cold Spring Harb. Symp. Quant. Biol. 83, 97–103. 10.1101/sqb.2018.83.038109 30948452

[B221] Zelikowsky M. Hui M. Karigo T. Choe A. Yang B. Blanco M. R.. (2018b). The neuropeptide Tac2 controls a distributed brain state induced by chronic social isolation stress. Cell 173, 1265–1279.e19. 10.1016/j.cell.2018.03.037 29775595 PMC5967263

[B222] Zernig G. Kummer K. K. Prast J. M. (2013). Dyadic social interaction as an alternative reward to cocaine. Front. Psychiatry 4:100. 10.3389/fpsyt.2013.00100 24062696 PMC3770939

[B223] Zorrilla E. P. Logrip M. L. Koob G. F. (2014). Corticotropin releasing factor: a key role in the neurobiology of addiction. Front. Neuroendocrinol. 35, 234–244. 10.1016/j.yfrne.2014.01.001 24456850 PMC4213066

